# Stable Diffusion Models Reveal a Persisting Human–AI Gap in Visual Creativity

**DOI:** 10.1002/advs.202524142

**Published:** 2026-03-24

**Authors:** Silvia Rondini, Claudia Alvarez‐Martin, Paula Angermair‐Barkai, Olivier Penacchio, Marc Paz, Matthew Pelowski, Dan Dediu, Antoni Rodriguez‐Fornells, Xim Cerda‐Company

**Affiliations:** ^1^ Cognition and Brain Plasticity Unit Bellvitge Biomedical Research Institute L'Hospitalet De Llobregat Spain; ^2^ Bridging Research in AI and Neuroscience Computer Vision Center Bellaterra Spain; ^3^ Department of Cognition Development and Educational Psychology University of Barcelona Barcelona Spain; ^4^ Vienna Cognitive Science Hub Vienna Austria; ^5^ Faculty of Psychology University of Vienna Vienna Austria; ^6^ Computer Science Department Universitat Autonoma de Barcelona Bellaterra Spain; ^7^ University of Barcelona Institute For Complex Systems (UBICS) Barcelona Spain; ^8^ Department of Catalan Philology and General Linguistics University of Barcelona Barcelona Spain; ^9^ Catalan Institution for Research and Advanced Studies (ICREA) Barcelona Spain; ^10^ Aix‐Marseille University Iméra Marseille France; ^11^ Institut de Neurociències (UBNeuro) Universitat de Barcelona Barcelona Spain

**Keywords:** creativity, generative AI, stable diffusion

## Abstract

While recent research suggests Large Language Models match human creative performance in divergent thinking tasks, visual creativity remains underexplored. This study compared image generation in human participants (Visual Artists and Non‐Artists) and using an image‐generation AI model (two prompting conditions with varying human input: high for Human‐Inspired, low for Self‐Guided). The creativity of the resulting images was evaluated by human raters (N = 255) and GPT‐4o acting as an AI rater under two conditions: strictly mirroring the human rating task and using in‐context learning with human‐rated examples as guidance. We observed a clear creativity gradient: Visual Artists > Non‐Artists ≥ Human‐Inspired GenAI > Self‐Guided GenAI. Increased human guidance strongly improved GenAI's creative output, bringing its productions close to those of Non‐Artists. Moreover, while Guided‐GPT‐4o more closely approximated human creativity judgment patterns, baseline GPT‐4o (without guidance) exhibited markedly different creativity evaluations, showing reduced discrimination between image categories and inflated scores for GenAI outputs. These results suggest that, in contrast to language‐centered tasks, GenAI models may face unique challenges in visual domains, where creativity depends on perceptual nuance and contextual sensitivity, distinctly human capacities that may not be readily transferable from language models.

## Graphical abstract

This study examines visual creativity in humans and generative AI using the TCIA framework. Human artists outperform AI overall, yet structured human guidance substantially improves AI outputs and evaluations. Findings reveal that alignment with human creativity depends critically on contextual framing, highlighting both the promise and current limitations of AI in visually grounded creative tasks.

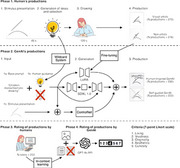

## 1 Introduction

The recent popularization of Generative AI GenAI) models, largely successful at mimicking human verbal and visual productions [1], have stimulated a growing body of research focused on their creative potential [2]. GenAI models have been dubbed “Artificial Muses” [3], “Engines of Wow” [4], “Socio‐Technical Monsters” [5], depending on the observer's placement on the AI utopia‐dystopia spectrum [6]. Large Language Models (LLMs) are GenAI systems designed for natural language processing and generation, typically pre‐trained on vast text corpora [7]. In the field of computational creativity, this class of models has now been evaluated largely through divergent thinking (DT) assessments [8, 9, 10]. DT is generally understood as the ability to form associations between semantically distant ideas, breaking away from prevailing modes of thought, leading to original solutions to open‐ended problems, with DT productions usually being assessed across dimensions of Flexibility, Fluency, Originality, and Elaboration [11, 12].

The existing research has repeatedly shown that LLMs may often outperform humans in DT [2, 13, 14], with the exception of a few studies where the most creative humans still outdid the models [3, 10, 15]. Furthermore, when it comes to creativity perception, LLMs have been employed to evaluate both human and GenAI DT tests’ responses and have been proven to provide accurate scores, with high correlation with the creativity judgements of human raters [3, 16]. Overall, considering these recent studies and the standardized definition of creativity as the process leading to the production of something both novel and useful [17], GenAI could indeed be regarded as a ‘creative agent,’ and a remarkably powerful one at that. Nonetheless, points of criticism around computational creativity have also emerged, from GenAI's reliance on statistical recombination of training data in its creative process to, most importantly, the issue of prompt‐dependency, arguably leading to a lack of true creative agency and intentionality in the models [3, 18, 19].

Another existing gap in our understanding of GenAI concerns visual creativity, and particularly idea generation or the imaginative process. While the vast majority of computational creativity studies have focused on language‐based DT tasks in LLMs, visual creativity in image‐generation models remains largely overlooked. A series of studies on one‐shot image generation across different GenAI models has evaluated their ability to mimic human sketch drawing without, however, specifically addressing creativity itself [20, 21, 22]. Another study examined GPT‐4o's creative interpretation of abstract visual stimuli; however, it only analyzed the model's verbal outputs according to DT parameters [23]. Overall, the unilateral focus of previous studies on language‐based GenAI and DT as the main dimension of creativity makes any broad claims regarding the creative advantages of computational agents premature.

Visual creativity, specifically in the form of visual imagery generation or imagination as an end in itself, is often regarded as a key counterpoint to its verbal dimensions [24]. From an evolutionary point of view, imagination has been linked to the inception of abstract thought, self‐idealisation, and consciousness as a whole, while also acting as a bridge between the sensory and cognitive dimensions, allowing us to mentally explore possible scenarios in the absence of direct sensorial stimuli [25, 26, 27]. Visual mental imagery (VMI), or “seeing with the mind's eye” [28, 29], engages neural networks spanning from perceptual vision, including early visual areas, to higher‐order elements from episodic and autobiographical memory [29, 30, 31, 32]. What we term “Creative Mental Imagery” (CMI) in this paper, however, refers to the internal creation of original images: a testament to the brain's capacity to synthesize new ideas and scenarios independent of direct perception. CMI has been shown to support our information‐processing through depictive representations [31], participating in our evolution as ever‐innovating, ever‐creative beings [33]. Despite the theory of DT having established itself as the dominant paradigm in creativity research [34], the involvement of mental imagery in creative processes is substantial [35, 36, 37, 38].

The embedded nature of visual imagination, spanning from perceptual to higher‐order, semantic abilities, becomes manifest in the complexity of its product, creative imagery, its scoring based on its physical properties, as well as its conceptual content [33]. Current DT studies have so far drawn conclusions on GenAI creative superiority based on verbal creative outputs, easily explained by the computational disparities between human and AI agents. CMI thus constitutes a particularly interesting strategy for the investigation of computational creativity from a more complex lens, to include perceptual and semantic skills, beyond the agents’ computational abilities.

Thus, our project focused on the visual imagination abilities of a Stable Diffusion model compared to human samples, by observing both production and appraisal of creative mental imagery. Stable Diffusion is a class of diffusion‐based text‐to‐image models, which generates images starting with random pixel noise in an image grid and iteratively refines it, using the text prompt at each step to guide the denoising toward features and patterns that match the prompt until a coherent image forms [21, 22]. At present, they constitute the class that best mimics human sketch‐drawing [22, 23] and were therefore selected for the project. Potential similarities and contrasts in the creative visual behavior of humans and GenAI may shed light on the important differences in how computational and human agents interact creatively with visual stimuli, both perceptually and semantically, thus offering a broader stance on creativity in state‐of‐the‐art models. Additionally, we address the underlying issue of intentionality and agency in the creative process of GenAI models by introducing different degrees of human guidance through prompting, aiming to determine the extent to which human input influences GenAI's successful creative outputs.

**FIGURE 1:**
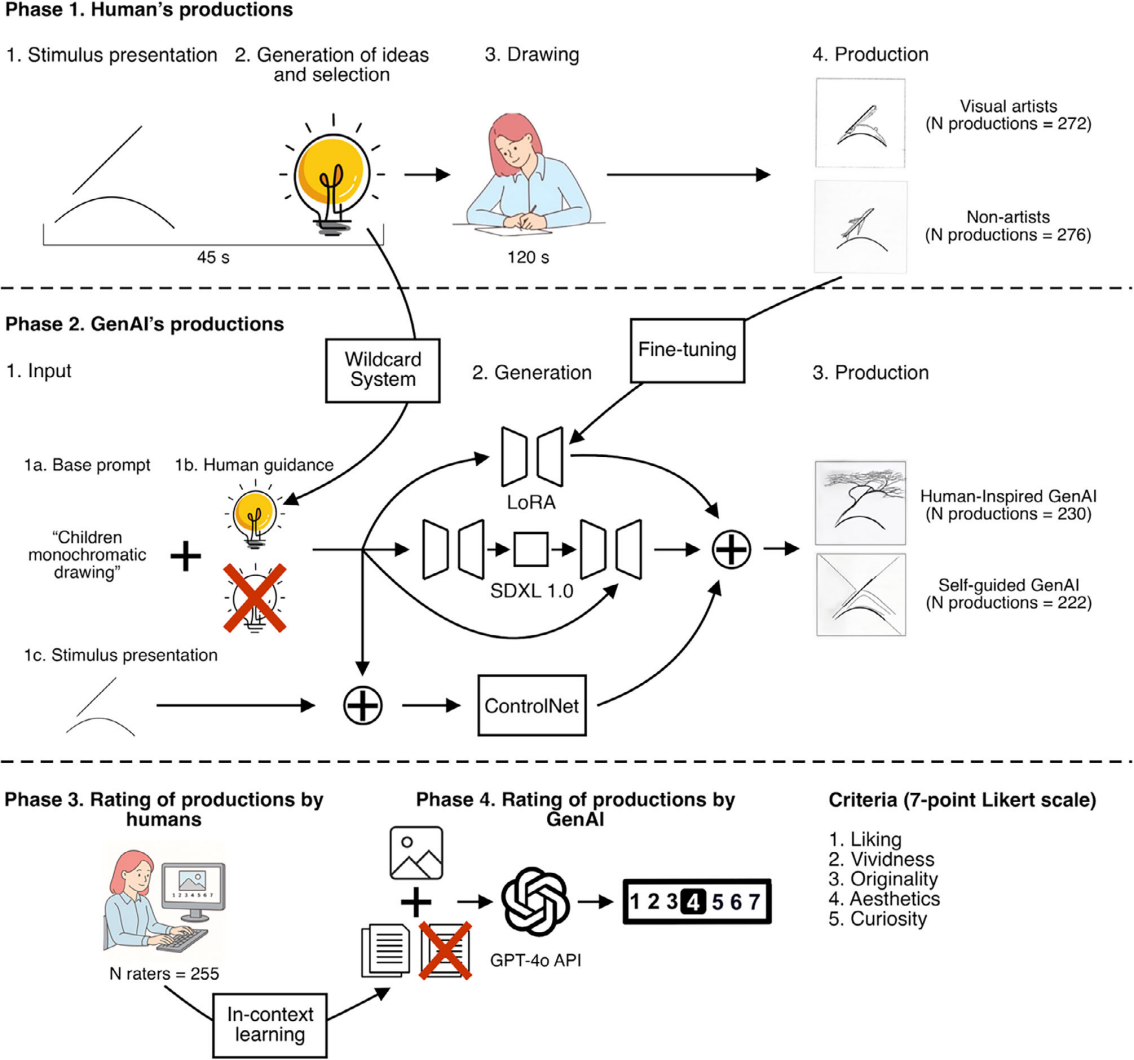
Scheme of the study's four phases. Phase I: Creative image generation by Visual‐Artists and Non‐Artists. The resulting human images were used to fine‐tune the Diffusion model (SDXL) through Low‐Rank Adaptation (LoRA), while the human‐generated ideas were used in the prompt of the Human‐Inspired GenAI group. Phase II: Creative image generation by GenAI (Human‐Inspired and Self‐Guided). Phase III: Creative image dataset rating by human raters. Phase IV: Creative image dataset rating by both GPT‐4o and Guided‐GPT‐4o (using in‐context learning).

The present study comprised four phases (for a scheme of the study, see Figure 1 and Materials and Methods for details). Phases I and II were based on the Test of Creative Imagery Abilities (TCIA) [34], and aimed at generating two distinct datasets of creative drawings: by humans, comprising visual artists (N = 27) and non‐expert participants (N = 26), and from GenAI models. Since previous studies on one‐shot image generation had determined Diffusion models to be the most proficient class of GenAI models at human drawing reproduction [20, 21, 22], we selected a Stable Diffusion model for our task [39].

In the TCIA, participants were presented with a series of 12 abstract stimuli, which they used to mentally generate images. Each participant then listed the images they had imagined and selected one to draw on paper. The TCIA was reproduced in the GenAI groups by employing the TCIA abstract stimuli as ControlNet and a basic prompt to generate drawings. As a key criticism of previous GenAI creativity research had targeted GenAI's lack of agency as pre‐emptive of a genuine creative process [3, 18, 19], different levels of prompt elaboration, and thus human guidance, were employed in the two GenAI groups of phase II.

For the Human‐Inspired GenAI condition, a more specific, concrete prompt containing one of the human‐generated ideas from phase I was employed, while the Self‐Guided GenAI condition employed the same base prompt, with the exception of the added human idea. Finally, phases III and IV focused on the scoring of the total generated dataset of 1,000 images, obtaining separate creativity ratings from both human and GenAI raters through an image rating task using five creativity dimensions (Liking, Vividness, Originality, Aesthetics, and Curiosity) [34].

In phase III, the image rating task was carried out online by human raters (N = 255). Prior to the image rating task, raters completed the Aesthetic Responsiveness to Art (AR eA) questionnaire [40] in order to determine whether differences in aesthetic tendencies led to variations in creativity judgments.

Each rater received a unique subset of 120 drawings, comprising an even distribution of images from all four experimental groups, while also ensuring that each image would gather a minimum of 30 ratings. No information was provided on the origin of the drawings. In phase IV, the same rating task was reproduced through the GPT‐4o API [41].

Due to the novelty of the study, our predictions were exploratory in nature. On one side, assuming that GenAI's linguistic competence generalizes to visual creativity, patterns similar to those of DT studies may be expected, with GenAI agents outperforming human participants [2, 13, 14], and with the standing possibility of the most creative humans still placing above the models [3, 10, 15]. Furthermore, based on previous findings showing refined prompt‐engineering techniques to successfully direct and enhance the creativity of GenAI outputs [42, 43, 44, 45, 46], it is likely that the GenAI group with increased human prompting will display higher creativity compared to the low‐guidance GenAI group.

When it comes to the appraisal of creative images, GenAI performance compared to humans remains relatively understudied, with the limited existing research showing weak aesthetic perception competence [47]. While some models do reach close correlations with human raters, the tasks usually focus on physical attributes of the images, rather than deeper dimensions of creativity [48, 49, 50]. Based on this, stronger similarities between human and GenAI raters may be expected to emerge for the more objective, computable creativity dimensions, with potentially more variation found for the more contextual, subject‐dependent features of a creative image.

## 2 Results

**FIGURE 2:**
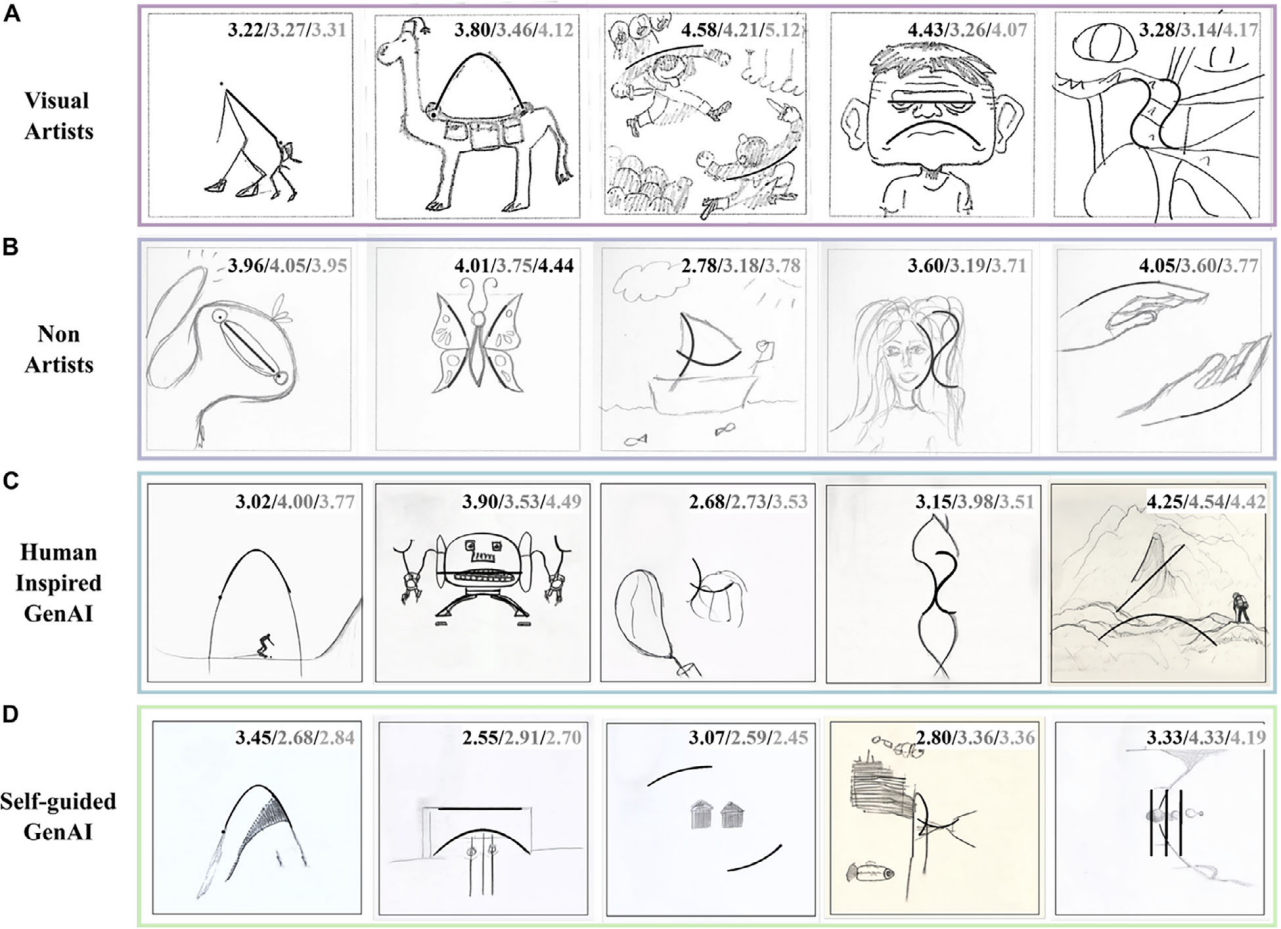
Examples of drawings from each category (in rows): Visual Artists, Non‐Artists, Human‐Inspired GenAI, Self‐Guided GenAI. The values reported in the right‐hand top corner of each drawing correspond to the drawing's Creativity score by humans, GPT‐4o, and Guided‐GPT‐4o raters, respectively.

Examples of the drawing outputs can be seen in Figure 2. The creativity ratings of the image dataset generated in phases I and II were obtained from human and GenAI raters in phases III and IV, respectively. These two sets of creativity ratings were analyzed first, individually, and then compared as follows.

### 2.1 Human Ratings: Analysis Results

#### 2.1.1 Overall Creativity Analysis

First, we evaluated the scoring reliability using a split‐half reliability approach, whereby the ratings were randomly partitioned into two halves 5,000 times, and the resulting 5,000 pairs of mean ratings were correlated. The resulting Spearman‐Brown corrected reliabilities across the five subscales ranged between 0.77 and 0.86 (Liking 0.83 ± 0.02; Vividness 0.86 ± 0.01; Originality 0.80 ± 0.02; Aesthetics 0.77 ± 0.02; Curiosity 0.77 ± 0.02), indicating good to strong reliability of image ratings.

For the analysis of human evaluations, an overall Creativity score was generated from the five rated dimensions (i.e., Liking, Vividness, Originality, Aesthetics, and Curiosity & Interest) using Factor Analysis (FA, see Materials and Methods for more details). An analysis dividing the sample between Human and GenAI images showed that, indeed, human‐generated images were scored significantly higher than GenAI ones (β = ‐.45, p <.001, see Table S1 for model details).

**FIGURE 3:**
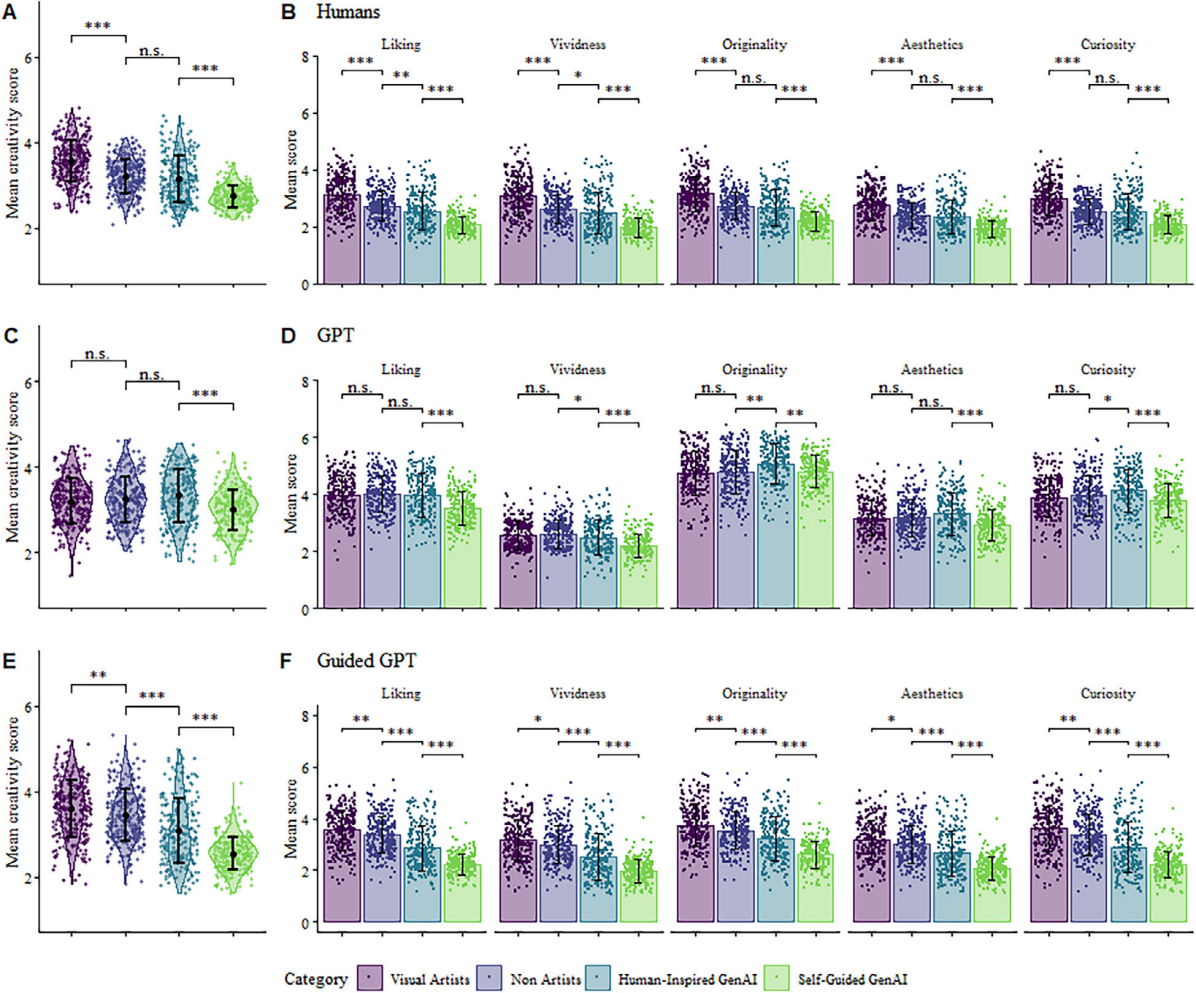
Creativity Ratings by Humans and GPT‐4o. A. Mean Creativity score (1–7) for each image category (Visual Artists, Non‐Artists, Human‐Inspired GenAI, and Self‐Guided)GenAI). B. Mean ratings for each Creativity dimension (Liking, Vividness, Originality, Aesthetics, Curiosity) by image category. C. Mean Creativity score for each image category based on GPT‐4o ratings. D. Mean ratings for each Creativity dimension by image category based on GPT‐4o evaluations. E. Mean Creativity score for each image category based on Guided‐GPT‐4o ratings. F. Mean ratings for each Creativity dimension by image category based on Guided‐GPT‐4o evaluations. Individual points represent image means, and error bars represent category mean ± standard deviation. Pairwise comparisons from linear mixed‐effect models are shown for adjacent categories. ^*^ *p* <.05, ^**^ *p* <.01, ^***^ *p* <.001. n.s. = not significant.

Subsequent analysis through linear mixed‐effects models (See Materials and Methods for models’ details) revealed image category to have an effect on the overall Creativity score (χ^2^(3) = 375.92, p <.001, ΔAIC = ‐369.92, see Table S2 for model details). Specifically, the Visual Artists group obtained the highest Creativity scores, followed by Non‐Artists images and Human‐Inspired GenAI, and lastly, Self‐Guided GenAI images (see Figure 3A).

Pairwise comparisons showed overall Creativity scores to be significantly different (*p* <.05) between categories (See Table S2): between Visual Artists and Non‐Artists (β = ‐.35, *p* <.001), between Visual Artists and HI‐GenAI (β = ‐.43, *p* <.001), and between Visual Artists and SG‐GenAI (β = ‐.83, *p* <.001). Importantly, the difference between Non‐Artists and HI‐GenAI groups was the only non‐significant one (β = ‐0.07, *p* = .052), with further complexities emerging when looking at individual dimensions of creativity (see below). This highlighted the marked effect of prompting the GenAI with human ideas on its overall creativity, leading to the model performing on par with non‐expert humans.

#### 2.1.2 Creativity Dimensions Analysis

The results from linear mixed‐effects models showed the same clear descending pattern between categories across all individual dimensions of Creativity (see Tables S3–S7). As reflected in Figure 3B, pairwise comparisons between categories were significant for all dimensions, with the exception of the comparison between Non‐Artists and HI‐GenAI in the Liking and Vividness dimensions.

In summary, these results indicate that images drawn by humans were rated as overall significantly more creative than those generated by GenAI. Within the GenAI group, incorporating human ideas into the prompt led to markedly higher creativity ratings, matching the scores of non‐expert humans.

### 2.2 GPT‐4o Ratings: Analysis Results

#### 2.2.1 Overall Creativity Analysis

To assess the effect of image category on the creativity scores in GenAI‐rated images, the same analysis performed on human ratings was repeated using the GPT‐4o and the Guided‐GPT‐4o ratings (see Figure 3C,E). On one hand, in GPT‐4o ratings, results from the linear mixed effects model suggested that, although the effect of category was significant (χ^2^(3) = 41.87, p <.001, ΔAIC = ‐35.87, see model summary in Table S8), this effect was driven by the SG‐GenAI category, which showed significantly lower scores than all other categories. Indeed, analysis comparing between Human and GenAI images showed no significant effect of group (χ^2^(1) = 2.08, p = .15, ΔAIC = ‐.08). On the other hand, in Guided‐GPT‐4o ratings, linear mixed‐effects models revealed image category to have an effect on the overall Creativity score (χ^2^(3) = 355.23, p <.001, ΔAIC = ‐349.23, see Table S10). Specifically, the Visual Artists group obtained the highest Creativity scores, followed by Non‐Artists images and HI‐GenAI, and lastly, SG‐GenAI images (see Figure 3E).

#### 2.2.2 Creativity Dimensions Analysis

As in the analysis of human ratings, linear mixed‐effects models were used to study the effect of image category on the remaining creativity dimensions for GPT‐4o (Tables S10–S14) and Guided‐GPT‐4o (Tables S15–S19). Consistent with the overall Creativity results, Guided‐GPT (but not GPT) exhibited a coherent and discriminative pattern across all Creativity dimensions (see Figure 3D,F). Specifically, Guided‐GPT‐4o followed a structured hierarchy across categories, although SG‐GenAI was rated significantly lower than the other categories across all dimensions.

Interestingly, in GPT ratings, SG‐GenAI images were likewise rated significantly lower than other categories across all dimensions. However, HI‐GenAI images received significantly higher scores in Originality and Curiosity, whereas the highest Vividness ratings were observed in the Non‐Artists category.

### 2.3 Similarities and Differences Between Human and GPT‐4o Creativity Ratings

**FIGURE 4:**
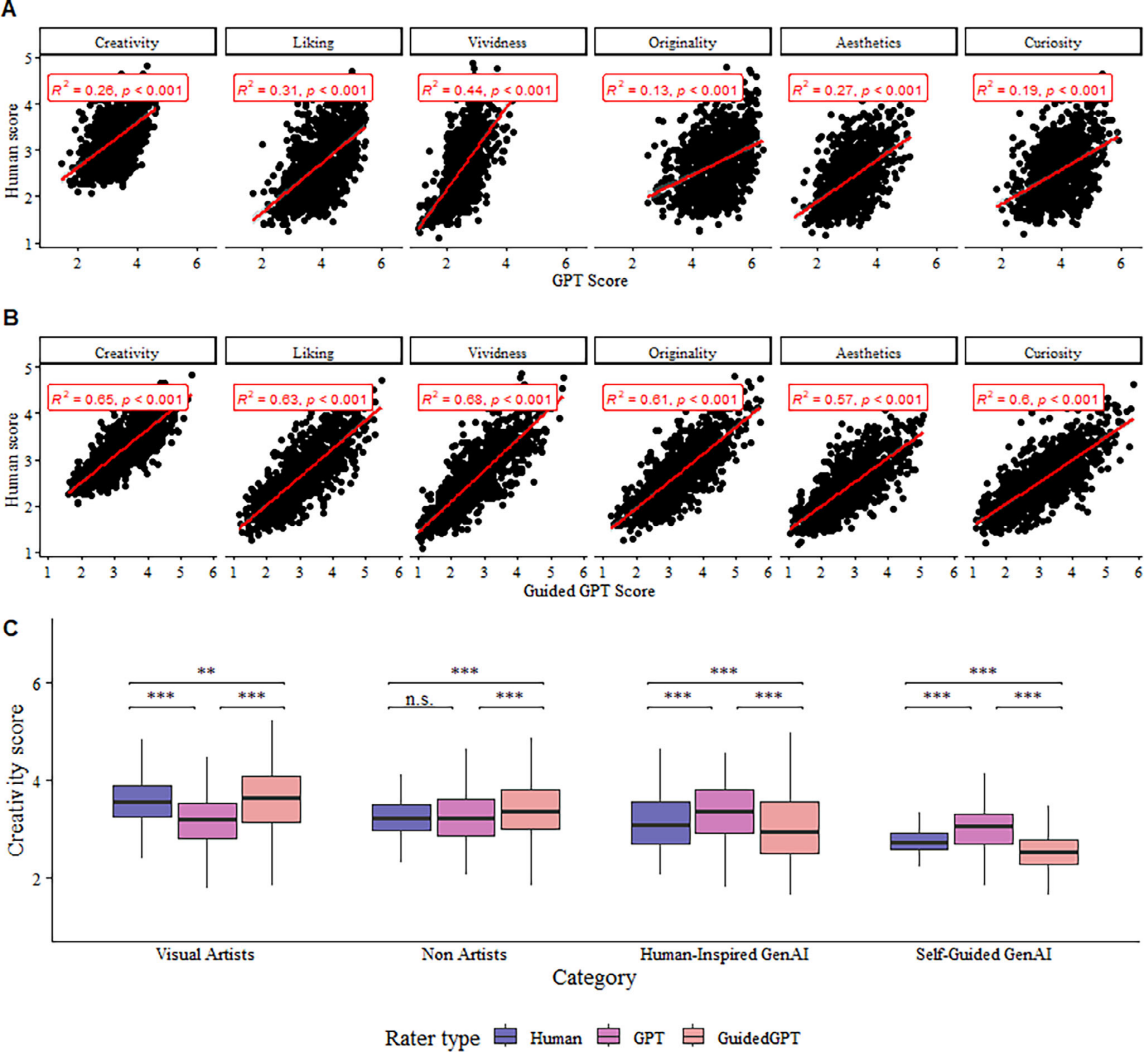
Similarities and differences between Human and GPT‐4o ratings. A. Pearson correlations between Human and GPT‐4o across the Creativity score and the five rated subscales. B. Pearson correlations between Human and Guided‐GPT‐4o ratings across the Creativity score and the five rated subscales. C. Boxplot showing Human vs GPT‐4o overall Creativity ratings across image categories (Visual Artists, Non‐Artists, HI‐GenAI, and SG‐GenAI).

To assess the agreement between Human ratings and those of GPT‐4o and Guided‐GPT‐4o, we computed the Pearson correlations between the Human‐GPT and Human‐Guided‐GPT pairs separately for each of the five subscales and for the overall Creativity score. Across all dimensions, the correlations between Human and GPT‐4o were positive and statistically significant, but weak (Figure 4A), ranging from 0.13 (p <.001; in Originality) to 0.44 (p <.001; in Aesthetics), while those between Human and Guided‐GPT‐4o ratings (Figure 4B) were also positive but substantially stronger across all subscales, ranging from 0.57 to 0.68; Fisher's r‐to‐z comparisons confirmed that they were significantly higher (all Zs ≤ −7.97; *p*s <.001).

Given the different patterns found for the Creativity rating of Human versus GenAI drawings, a new linear mixed‐effects model was fitted to test whether these differences were significant (see Table S20). Indeed, the interaction between image category and type of rater was confirmed to be significant (χ^2^(6) = 2,741.23, *p* <.001, ΔAIC = ‐2,729.23), indicating different ratings patterns between humans, GPT‐4o, and Guided‐GPT‐4o.

Specifically, human raters assigned the highest creativity scores to images produced by Visual Artists, followed by progressively lower scores for Non‐Artists, HI‐GenAI, and SG‐GenAI images. Guided‐GPT‐4o ratings mirrored this same hierarchical pattern, with Visual Artists’ images receiving the highest scores, followed by Non‐Artists, HI‐GenAI, and SG‐GenAI. In contrast, GPT‐4o exhibited a markedly different pattern (see Figure 4C). GPT‐4o ratings showed increased creativity judgments across all image categories, while displaying less discrimination between them, in some cases even favoring GenAI images. The most pronounced differences between human and GPT‐4o ratings emerged for the HI‐GenAI and SG‐GenAI images. Whereas human raters assigned these categories the lowest overall Creativity scores, GPT‐4o rated them at levels comparable to (or even higher than) those given to human‐generated images. These patterns highlighted a tendency in the GPT‐4 orator to assign more generous creativity scores, particularly in the case of GenAI images, strongly contrasting with the human ratings, which were overall more conservative and showed significant discrimination between image categories.

**FIGURE 5:**
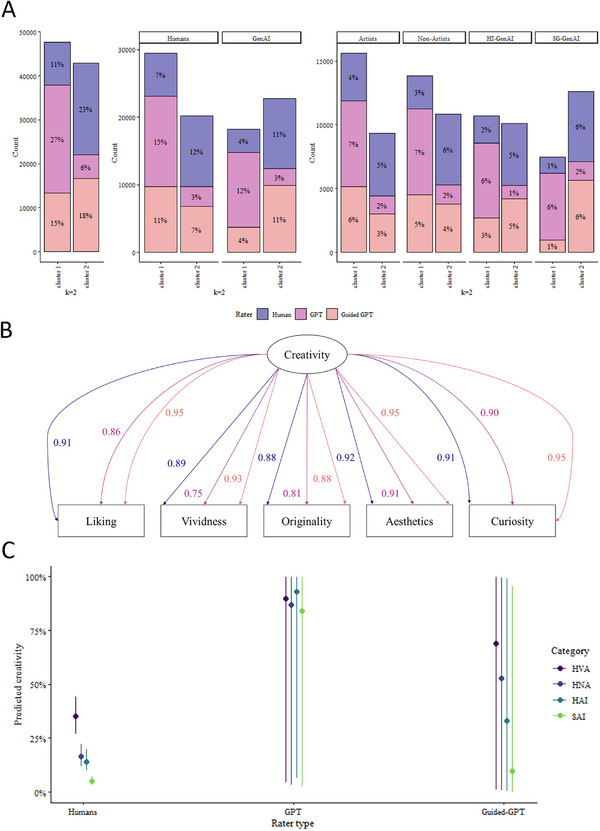
A. Distribution of the responses among the k‐means with k = 2 clusters. Left: by rater type (colour, Human vs. GPT‐4o vs. Guided‐GPT‐4o). Middle: by rater type (colour) and drawer type (Human or Stable Diffusion). Right: by rater type (colour) and image category (Non‐Artists, Visual Artists, Human‐Inspired GenAI, and Self‐Guided GenAI). B. The one‐factor model fitted to the whole dataset, split by rater type (Human vs. GPT‐4o and Guided‐GPT‐4o). There are three arrows and loadings for each indicator, the first being for the human raters, the second for the GPT‐4o rater, and the third for the Guided‐GPT‐4o rater. The loadings are standardized, and all of them were significant (all *p*s <.001). C. Predicted Creativity score from a beta regression on the interaction between rater type and image category while controlling (as random effects) for item and individual rater.

Moreover, the distribution of the responses on the five dimensions (see Figure S1) already hints that human and GPT‐4o may be fundamentally different, with the Guided‐GPT‐4o falling somewhere in between these two, which is further confirmed by their *k*‐means clustering. We systematically analyzed all clusters between *k* = 2 and *k* = 6, and we found that the main division (*k* = 2; see Figure 5A) was between a cluster of mostly human (23% vs. 11%) and Guided‐GPT‐4o (18% vs. 15%) ratings, and another of mostly GPT‐4o ratings (27% vs. 6%), while higher *k* values further refined these two clusters.

The five dimensions had 11.2% Guttman errors bigger than (Q3 + 1.5*IQR) = 7.5, and a set homogeneity value *H* = 0.831 with 95% confidence interval (0.828, 0.833), which was significantly higher than the recommended 0.30, suggesting that the scale was homogeneous; this was further supported by the fact that all items had a homogeneity ≥ 0.750. Likewise, the Automatic Item Selection Procedure (*aisp*) found that all dimensions belonged to the same scale for all *c* between 0.05 and 0.60. A Principal Component Analysis (PCA) of all the ratings together found that the first principal component (PC_1_) explained 82.5% of the variance, and all five dimensions had loadings of the same sign and comparable strengths. When the analysis was performed separately for Human, GPT‐4o, and Guided‐GPT‐4o ratings, PC_1_ explained 84.9%, 77.0%, and 89.1% of the variance, respectively (see Figure S2A,C,E). Across groups, the general loading structure appeared similar (see Figure S2B,D,F), although minor differences in loading magnitudes were observed (see Figure S2D). These results were supported by an exploratory Factor Analysis (EFA), with the one‐factor model explaining 78.6% of the variance when all judges were pooled together, 81.8% when only human judges were considered, 71.9% for GPT‐4o, and 86.5% for Guided‐GPT‐4o. Fitting a one‐factor confirmatory multi‐group (by judge type) Factor Analysis (CFA) model (see Figure 5B) resulted in a significant *p*‐value (*χ* ^2^(15) = 28,312.84; *p* <.001), but with decent fit indices (CFI = 0.94, TLI = 0.89 and NNFI = 0.89), which was not surprising given the amount of data and the complexity of the design. Interestingly, checking its configurational, weak and strong invariance, strongly suggested that both the factor loadings (*χ* ^2^(8) = 4,907; *p* <.001) and the latent means (*χ* ^2^(8) = 94,935; *p* <.001) were different between Human, GPT‐4o and Guided‐GPT‐4o ratings, with the inspection of the loadings (Figure 5B) suggesting that Guided‐GPT‐4o ratings were more similar to human judgments than to the GPT‐4o ones. From this model, we estimated the latent “Creativity score” (using the loadings for Humans separately, GPT‐4o and Guided‐GPT‐4o), which was our outcome of interest in the following regression models.

In a series of mixed‐effects beta regression models predicting creativity from rater type, drawer type, and category, we found no significant main effect of rater type (*p* = .99), but a significant interaction with drawer type (*p* <.001) and with the category (*p* <.001; see Figure 5C). That is, (a) while the humans made a clear distinction between kinds of drawings, with a clear hierarchy Visual Artists ≫ Non‐Artists ≳ Human‐Inspired GenAI ≫ Self‐Guided GenAI (the first and last highly statistically significant), GPT‐4o did not, with Guided‐GPT‐4o showing the same pattern as the humans in terms of point estimates, but with wide confidence intervals precluding any statistically significant differences, and (b) while the error bars of the human raters were very constrained, those for GPT‐4o and Guided‐GPT‐4o were notably wider. Taken together, these findings suggested that while on average Human, GPT‐4o, and, in particular, Guided‐GPT‐4o scores were similar, this may be misleading as the human scores are rather focused and in agreement with each other for a given image category, while GPT‐4o's and Guided‐GPT‐4o's were scattered across pretty much the whole range of possible scores.

The visual stimulus used to prompt a drawing had a main effect and interacted significantly with the rater type. However, in practice, this resulted in minimal changes in the scores that did not affect the qualitative results (see Table S21 for model details).

**FIGURE 6:**
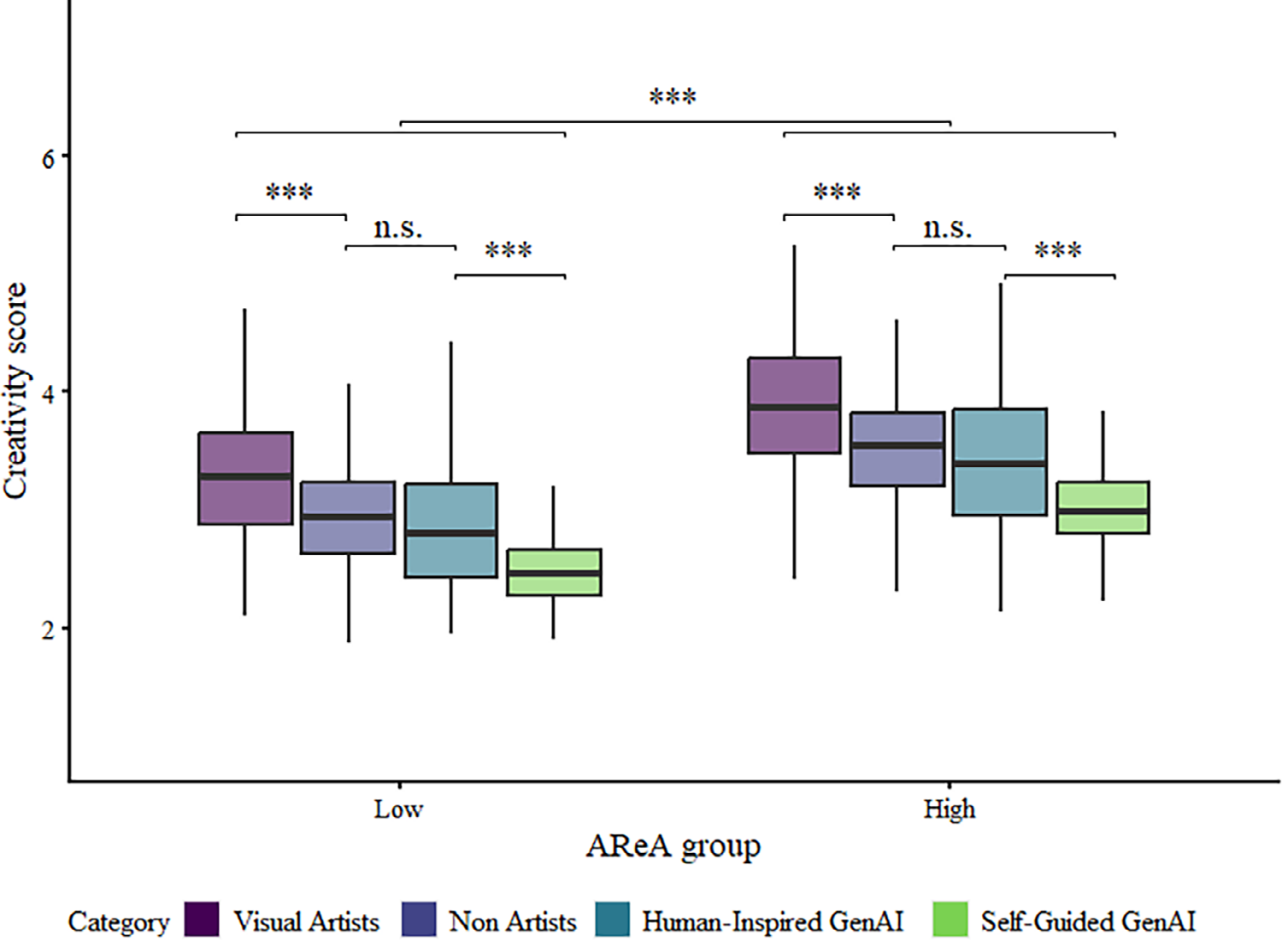
Impact of AReA scores on Creativity ratings. High‐AReA participants assign overall higher creativity ratings than low‐AReA participants. The pattern of discrimination between image categories remains constant.

Focusing on the human raters, individuals were divided into Low and High AReA groups using a median split (AReA = 2.93). The main factor influencing their evaluations was their own AReA score. A significant positive main effect was found (χ^2^(4) = 1,721.23, p <.001, ΔAIC = ‐1,713.23; see Figure 6 and Table S22 for model details), indicating that individuals with higher AReA scores reported higher Creativity ratings than those in the Low AReA group. However, in this case, the interaction was not significant (χ^2^(3) = 1.51, p = .68, ΔAIC = 4.49), with analogous rating patterns between the two groups.

## 3 Discussion

The study explored visual creativity in image‐generation models and humans through the Test of Creative Imagery Abilities (TCIA). To allow comparison with a larger range of human performance at the TCIA, a group including both Visual Artists and Non‐Artists was recruited. Furthermore, in order to investigate issues of prompt‐dependency and their impact on computational agents’ creative performance, different degrees of human guidance through prompt‐elaboration (HI‐GenAI and SG‐GenAI) were introduced.

The creativity of uman and GenAI images was scored by both human and AI raters. The AI raters were simulated by GPT‐4o under two conditions: first, strictly mirroring the human rating task; and second, within an in‐context learning framing (Guided‐GPT‐4o), in which images paired with human ratings were provided as reference examples, mirroring the elaborate prompting condition of the HI‐GenAI group.

Results revealed a clear human advantage at the TCIA, with the Visual Artists’ productions being rated as the overall most creative, followed by the Non‐Artists’ and HI‐GenAI with comparable scores and, lastly, SG‐GenAI (see Figure 3A). Although the GenAI groups generally performed considerably worse than the human groups, providing a more elaborate, human‐inspired prompt elevated the HI‐GenAI group's outputs to human‐level performance.

When comparing creativity ratings, the baseline GPT‐4o rater did not replicate the human appraisal pattern: Neither overall creativity scores, nor image category discrimination matched between Human and GenAI ratings (see Figure 4C). In contrast, under the in‐context learning condition, Guided‐GPT‐4o's exhibited creativity rating patterns that more closely resembled those of human raters.

The major differences observed in GenAI visual creativity performance in our study stand in stark contrast with existing research, where GenAI consistently surpassed humans in verbal divergent thinking (DT) tasks. In previous computational creativity studies, generative algorithms generally performed well on Flexibility, Elaboration, and Fluency, with sometimes lacking or average results in Originality [3, 15]. While these results make intuitive sense due to GenAI's natural computational advantages in the generation of high numbers of elaborate, semantically flexible ideas, they also highlight core issues of employing DT tests as the principal computational creativity measure. By overly focusing on creativity dimensions such as Fluency, Flexibility, and Elaboration, heavily dependent on computational power and training‐data size, the obvious inequalities between Human and GenAI agents lead to the conclusion that computational agents outperform human creativity.

The highly specific structure of the queries in DT tests may constitute a further advantage for GenAI agents: clear and constrained prompting is a major factor in the optimal functioning of LLMs, while these same conditions may not be as encouraging of human creativity [34, 51]. Current architectures, functioning on closed‐world formalisms, excel at finding optimal solutions to well‐defined problems, while still, however, struggling when faced with the uncertainty constituted by the emergent possibilities and outcomes of open‐world problems [52]. Additionally, previous research has shown that humans tend to achieve higher Originality scores in figural DT tests compared to verbal ones [51]. Figural DT tasks tend to bypass the obvious, overlearned associations favored by verbal tasks, resulting in lower Fluency but higher Originality [51]. The structure of figural DT tests strongly resembles that of the TCIA, based entirely on abstract, non‐semantic stimuli. Accordingly, the open‐ended nature of our task could partially explain why, in our study, human participants outperformed GenAI across all creativity dimensions, in strong contrast with previous studies relying on verbal DT tasks, where specific, semantically loaded prompts favored GenAI performance, while potentially constraining human creativity.

On the other hand, the inclusion of a human‐generated idea in an otherwise basic, abstract prompt succeeded in elevating the creative results of the HI‐GenAI group, nearing the performance level of human Non‐Artists. Previous studies have argued that despite its creative potential, GenAI remains restricted as it does not experience the spontaneous need to ideate, to imagine, in the way human cognition does, relying on human input to simulate a real‐world connection in which that creative potential can be instantiated [3, 53]. GenAI agents are likely inherently solipsistic, existing and acting in a “small world” in an entirely predefined and formalized manner, their framing being constituted by the training dataset and the model's architecture [52, 54, 55]. Unlike human agents, their understanding of the outside world is not built through interaction, but rather through pure probabilistic associations gathered during training [56]. Human cognition, on the other hand, evolved in the context of maximizing a biological agent's adaptation and survival in an uncertain world, requiring a high degree of openness and generalization abilities [52]. Human creativity shares the same strong evolutionary roots as the ability to bring forth valuable and novel solutions to ever‐changing, open‐world problems [57, 58, 59]. It is thus largely relational, contextual, and sensitive to outer world conditions, allowing for flexible, adaptive responses to a complex environment [52, 60]. Human creativity, as defined by a novel and useful production, whether emerging through DT or imagination tasks, does not exist in a vacuum, but is rather a process instantiated between an embodied agent in an ongoing interaction with its environment [61]. In the case of GenAI agents, what prompting likely does is to provide the real‐world connection they lack to generate a contextually useful, creative product.

In contrast with DT tasks’ queries, highly specific and semantic in nature, the abstract visual stimuli of the TCIA constituted an extremely open‐ended, vague framing, which, when complemented by equally vague and open‐ended prompting, resulted in clearly lacking and uncreative productions in the SG‐GenAI group. The TCIA stimulus alone failed to trigger any creative image generation, as it would have in a human agent, who is able to leverage their biological memory and perception, as well as real‐world contextual information, to spontaneously and flexibly create [21, 62]. The addition of a concrete, human‐generated idea in the HI‐GenAI group's prompt essentially simulated in the model the creative process taking place in the human participants when exposed to the TCIA stimuli, considerably improving the creativity ratings of the productions. The performance gap between the SG‐GenAI and the HI‐GenAI groups resulting from more directed prompting underscores the importance of human guidance for successful GenAI creativity, while also showing that, at present, GenAI models cannot be regarded as autonomous creative agents.

Furthermore, the drastic differences between Human and GenAI raters at the image rating task (see Figure 4C) strongly contrasted the studies that had found LLMs to constitute accurate raters of verbal DT responses, highly correlating with human ratings [3, 16]. Despite the variance in aesthetic tendencies among the sample of human raters (see Figure 5C), the creativity appraisal patterns remained consistent across the different experimental categories, with a clear descending trend from the Visual Artist group to the SG‐GenAI group. In contrast, the baseline GPT‐4o ratings showed no clear discrimination between image categories and inconsistent patterns between the individual creativity dimensions.

Crucially, the Guided‐GPT‐4o condition, implemented via in‐context learning, noticeably reduced this discrepancy. When provided with reference human images and associated ratings, GenAI creativity rating patterns more closely matched those of human raters. This mirrors the earlier finding that HI‐GenAI outperformed SG‐GenAI: in both cases, performance improved when models were paired with explicit and structured human guidance. Just as the inclusion of a human‐generated idea in the HI‐GenAI prompt provided a conceptual structure for image production, the set of reference human images and ratings in the Guided‐GPT‐4o condition constituted a perceptual and semantic “frame” that GenAI could use to approximate human‐like aesthetic judgements. These results suggest that alignment with human creativity patterns is facilitated not merely by model scale, but by the presence of structured human‐derived contextual signals.

The dynamics underlying GenAI's difficulties in reproducing the human creative process may also explain the gap in visual creativity appraisal. Text and images are processed in fundamentally different ways by GenAI models: text is sequential and symbolic, thus easily captured by the probability‐based relationships on which transformer architectures rely [52, 63]. Specifically, the scoring of DT test responses by LLMs rests heavily on their number, elaboration, and their content's semantic distance, all easily quantifiable and computable dimensions [64]. On the other hand, images, creative or not, are high‐dimensional and spatial, requiring models to understand complex visual structures, leading to additional perceptual challenges arising in the rating of creative TCIA drawings. The complexities of visual perception increase in the appraisal of visual creative products beyond physical features, their creativity being largely determined by the observer's inner psychological state and outer socio‐cultural context [65]. These intrinsic complexities of visual perception and evaluation likely contribute to discrepancies observed between human and model‐based creativity assessments.

Consistent with this interpretation, Pearson correlations between Human and GPT‐4o ratings across creativity dimensions revealed the highest agreement for Vividness, followed by Liking and Aesthetics, with Curiosity and Originality showing the weakest correspondence (Figure 4A). Fisher's r‐to‐z test confirmed significant differences between Vividness and Liking (Z = 3.39; p <.001), Liking and Curiosity (Z = 2.86; p = .004), and Aesthetics and Originality correlations (Z = 3.26; p = .001).

In contrast, correlations between Human and Guided‐GPT‐4o ratings were consistently stronger across dimensions (Figure 4B), significantly exceeding those observed for GPT‐4o alone. Although the ordering of agreement was slightly different, the strongest correspondence was again observed for Vividness, followed by Liking, Originality, Curiosity, and finally Aesthetics. Thus, while in‐context guidance substantially increased overall alignment with human judgments, some variability across dimensions remained.

This pattern may reflect the nature of GPT‐4o's multimodal training, which primarily aligns images with descriptive text drawn from large web‐scale and proprietary corpora. As a result, the model learns associations between visual features and linguistic descriptors, but not explicit evaluative constructs such as Liking, Aesthetics, Originality, or Curiosity, as its objective emphasizes semantic and perceptual alignment rather than subjective or creative judgment [66]. Accordingly, dimensions more directly grounded in perceptual features and descriptive language (Liking and Vividness) exhibit greater structural correspondence, whereas dimensions requiring contextual, relational, and expectation‐based appraisal (e.g., Originality and Curiosity) show reduced agreement between Human and GPT ratings. The latter constructs depend on prior knowledge and socio‐cultural context, which are not directly encoded in image–text alignment objectives, providing a plausible explanation for the selective convergence observed across dimensions.

The marked increase in correlation strength under the Guided‐GPT‐4o condition suggests that providing human reference images and ratings offers an evaluative frame that partially compensates for these limitations. By incorporating explicit examples of human aesthetic judgements, the model is able to better approximate the contextual and relational criteria underlying human creativity appraisal, thereby reducing (but not fully eliminating) the discrepancy observed in the GPT condition.

In essence, the characteristics of multimodal LLMs’ training [66], in concomitance with their fundamentally different perceptual strategies and lack of contextual awareness [21, 52, 53], while not particularly impacting language processing, may play a larger role in highly complex and subjective processes such as creativity judgment. The different patterns emerging from GPT‐4o and human ratings solidify the stance that computational agents’ visual creative behavior does not yet match that of humans, its functioning likely relying on evolved biological characteristics and continuous adaptive interaction with the open environment, features that have not, at present, been successfully reproduced in GenAI models.

Overall, in both tasks, Stable Diffusion and GPT‐4o's required highly specific and directed human input to reproduce human‐level performance, thus reconfirming the stance that, while technically promising, current models are not capable of independent creative behavior.

### 3.1 Limitations of the Study

The current study focuses solely on Stable Diffusion as a class of generative AI systems, leaving aside the more recent multimodal LLMs. While the latter do exhibit advanced image‐generation abilities, it was impossible, at the time of the study, to replicate the same controlled experimental conditions that could be implemented in Stable Diffusion via LoRA fine‐tuning and ControlNet conditioning. Commercial multimodal LLMs are closed‐source and, therefore, cannot be tuned to produce outputs that are stylistically and structurally comparable to human drawings without providing overt guidance through human examples in the prompts, which would prevent an unbiased creative process. This class of models would therefore necessitate the formulation of a fundamentally different methodology, which lies beyond the scope of the current study.

It should also be mentioned that the TCIA [34] predates the release of Stable Diffusion XL Base 1.0 [39], and the exact content of its training dataset is undisclosed, making it impossible to verify a potential previous exposure of the model to the TCIA stimuli. However, due to the size of training datasets of text‐to‐image models, usually in the order of billions of image‐text pairs [67], it is highly unlikely that any potential data leakage from the TCIA could have biased the model's performance in any significant way. Moreover, only half of the stimuli employed were part of the original TCIA; the other half had been newly generated specifically for this task, and no express mention of the TCIA was made in the prompts employed in the study. The model's outputs were further constrained by the ControlNet conditioning and LoRA fine‐tuning. Inputs to the model did not include dataset identifiers or metadata, and images were synthesized de novo in response to task‐defined stimuli. Nonetheless, we acknowledge that potential pretraining exposure remains a general limitation of foundation models.

## 4 Conclusions

The present study probed the current state of GenAI visual creativity by directly contrasting human and GenAI performance in the production and evaluation of creative images. Using a vision‐based imagination task, rather than standard verbal tests, we showed that GenAI remains markedly limited in creative capacity, underscoring the need for broader measures to better triangulate the complexity of creativity in computational agents. The challenges GenAI faces in visual creativity could stem from fundamental divergences with human cognition and perception, including a reduced sensitivity to real‐world complexity. The improvement observed in both tasks under human guidance hints at the deeply embedded nature of human creativity, as an evolutionary process at the cusp between a biological agent and the complex, dynamic environment it inhabits, and points to a fundamental limitation in current GenAI. Overall, the models’ reliance on human prompting indicates that, while GenAI can exhibit creative abilities when properly guided, it does not yet reach autonomous human‐level performance.

## 5 Materials and Methods

### 5.1 Materials

An adapted version of the TCIA [34] was employed, where each participant is presented with a sequence of 12 abstract stimuli. Six out of the 12 stimuli were taken from the original TCIA, while the remaining six were newly created (see Figures S3 and S4). For the rating of our dataset, to the original TCIA scoring criteria “Vividness” and “Originality” [34], creativity measures of “Liking”, “Aesthetics”, and “Curiosity” were added (see Section S1 for detailed creativity measures selection).

The studies involving human participants, both in‐lab and online, were performed in accordance with the Declaration of Helsinki and approved by the Clinical Research Ethics Committee of the Bellvitge University Hospital (PR335/21). Participants received monetary compensation, and they provided informed consent for their participation in the study.

### 5.2 Phase I. Creative Image Generation in Human Artists and Non‐Artists

This in‐lab study examined two distinct groups (Visual Artists and Non‐Artists) to investigate human performance in CMI, and to assess potential differences in creative imagination abilities between individuals who routinely engage in CMI‐based activities (Visual Artists) and the general population (Non‐Artists; see Figure 1). Participants from both groups were economically compensated for their participation. The groups were composed, respectively, of:

1. Human Visual Artists (n = 27; mean age = 25.4 ± 4.8 y.o.; 14 female, 13 male), recruited from local fine arts institutes and ateliers (mean years practicing visual arts = 6.03 ± 3.95). Each participant was required to provide proof of enrolment in, or completion of, a visual art‐related degree or, alternatively, their professional art portfolio. Overall, participants’ nationalities included a total of nine countries and 15 different art schools, both within Spain and abroad, their degrees spanning across a wide range of visual arts disciplines.
2. Human Non‐Artists (n = 26; mean age = 25.2 ± 4.04 y.o.; 19 female, 7 male). Participants were recruited via Sona Systems (Sona Systems Ltd., Tallinn, Estonia), which resulted in a highly diverse sample, both in terms of academic and professional fields, and nationality, including participants from 13 different countries.

Overall, our sampling ensured that both groups included participants from multiple countries and backgrounds, reflecting broad individual diversity among the human groups.

### 5.3 Procedure

The TCIA was administered to human participants using Psychopy [68]. Participants were not informed of the task's aim, and no mention was made of creativity or imagination. The TCIA stimuli were presented in a randomized order. Each stimulus was shown individually for 45 s, and participants were asked to use it as a base to mentally generate any number of possible mental images (Figure 1). After each stimulus presentation, participants listed all the ideas they conceived before selecting one to reproduce on a provided sheet of paper, presenting the stimulus they had just observed. A maximum of 2 min was allocated for the completion of each drawing. A total of 660 images were generated: 324 by the Visual Artist group, and 312 by the Non‐Artist group.

### 5.4 Phase II: Creative Image Generation With GenAI—Stable Diffusion Models

This experiment aimed at reproducing the TCIA in a Stable Diffusion model, enabling a comparison between GenAI creative imagination with humans (See Figure 1). Two sets of GenAI drawings were produced, varying in the degree of human intervention through the elaboration of prompts:

1. Human‐Inspired (HI)‐GenAI: human ideas generated by the human groups were added to the basic prompt (see below).
2. Self‐Guided (SG)‐GenAI: no human ideas were added to the basic prompt (see below).

### 5.5 Procedure

GenAI images were generated employing a specifically fine‐tuned Stable Diffusion XL Base 1.0 [39] (See Section S2 for details on fine‐tuning and image generation). Each TCIA stimulus (see Figures S3 and S4) was employed as ControlNet input, paired with the base prompt “Children monochromatic [idea] drawing”, where [idea] (e.g., “whale”, “bridge”, “camping tent”) was randomly selected from human responses from Phase I, using a Wildcard system. The SG‐GenAI category used the same base prompt structure without the added [idea].

### 5.6 Final Dataset

The final dataset consisted of 1,000 images divided into four categories, as follows: Drawings generated by Human Visual Artists: 283; Drawings generated by Human Non‐Artists: 265; Drawings generated by HI‐GenAI: 230; and Drawings generated by SG‐GenAI: 222.

Images containing text, pseudo‐text, or colours were eliminated from the final dataset.

### 5.7 Phase III: Creative Image Rating of Image Dataset by Human Raters

To assess the creativity of the 1,000 generated images, an image rating task was designed and conducted through the online recruitment platform Prolific (See Figure 1). A total of 255 raters (Mean Age = 38.1 ± 12.7 y.o.; 126 female, 124 male, 5 Other; Mean Years of Education = 15.9 ± 4 years) took part and received an economic compensation for their participation.

All human raters were blind to the purpose of the study and were not informed of the origin of the images, nor did they receive any metadata or cues concerning GenAI models. A subset of the images was employed to carry out a Turing Test‐style discrimination task [69], which our architecture effectively passed (see Section S4 for Turing Test Methods and Results), making it unlikely that even if naïve human raters had formulated inferences around the drawings’ origin, their accuracy in doing so would have led to a significant bias toward either category of drawings.

To ensure an equal number of ratings for each of the 1,000 images, 250 unique subsets of 120 simple pencil drawings each were generated. Each subset contained a balanced distribution of images from each of the four experimental categories, while also ensuring that each image would appear a minimum of 30 times across the 250 sets. During the rating task, the images were presented to participants in a randomized order, and raters were told to rate them on five criteria. Each drawing was shown individually and was rated using 7‐point Likert scales from 1 (Not at all) to 7 (Extremely) across five dimensions: “Liking”, “Vividness”, “Originality”, “Aesthetics”, and “Curiosity & Interest”, presented in the same order for all raters (see Section S3).

The raters were first asked to complete the AReA questionnaire, a 14‐item scale designed to assess an individual's responsiveness to aesthetic experiences of diverse nature [40]. This allowed us to identify any potential impact of the individual raters’ aesthetic sensitivity on creativity scores and whether it led to different rating patterns emerging across each category of drawings.

### 5.8 Phase IV: Image Dataset Rating by GenAI Raters

Two further sets of ratings of the image dataset were carried out using GPT‐4o, both under a neutral prompting condition and an in‐context learning condition, in order to identify potential differences and similarities emerging between Human and GenAI agents in their appraisal of visual creativity (See Figure 1). The GenAI ratings of the database were carried out through the GPT‐4o API, by simulating the conditions of the human image rating task through a script. The LLM was prompted to behave like a human rater taking part in an online image rating task, receiving identical scoring instructions to the human raters (see Section S3). Each image was run through the GPT‐4o API 30 times to obtain the same number of ratings as the human sample. As previously mentioned, the first GenAI rating was carried out without any human guidance, replicating the exact conditions of the human rating task. In the GenAI rating with in‐context learning (Guided‐GPT), the model was provided with a randomly selected set of four reference drawings, one from each category, each paired with a corresponding human rating, selected at random from the 30 human ratings each image received in the human rating task. For each of the 30 GenAI rating repetitions per image, a new set of reference drawings and associated human ratings was randomly selected, resulting in unique contextual prompts for each of the 1,000 images.

Similar to human raters, GPT‐4o received identical instructions for all images and was not provided with any information about image provenance. Thus, all ratings were based exclusively on image content rather than inferred authorship.

### 5.9 Statistical Analysis

The dataset comprised 90,600 ratings of the 1,000 drawings (30,600 by human participants, 30,000 by GPT‐4o, and 30,000 by Guided‐GPT), containing, for each rating, the following information: the drawing's unique ID, the TCIA stimulus ID, the drawer type (Human or GenAI), the category (Visual artists, Non‐artists, HI‐GenAI and SG‐GenAI), the rater's unique ID, the rater's type (Human, GPT‐4o, or Guided‐GPT‐4o), and the scores on the five dimensions. For human raters, the dataset also included their age, gender, education level, and AReA score. For human drawers, we also included the drawer's unique ID, age, gender, education, AReA score, and, for the artists, their field of practice and number of years of practice.

Due to the experimental design, the resulting rater‐by‐image matrix was highly sparse, with most participant pairs sharing only a small number of rated images. To estimate the reliability of image‐level scores, we employed a split‐half reliability approach. For each image and rated dimension, the 30 ratings were randomly partitioned into two halves, and the mean ratings of these two subsets were correlated. This procedure was repeated 5,000 times to generate a distribution of reliability estimates and corresponding confidence intervals. All reliability coefficients were subsequently corrected using the Spearman‐Brown formula.

On this dataset, we applied various psychometric techniques [70] to the five dimensions and their scores. In particular, we employed *k*‐means clustering of the scores, correlation matrices with hierarchical clustering of the dimensions, Mokken Scaling Analysis looking at the distribution of the Guttman errors and the homogeneity values *H* of the whole set and of each dimension, as well as the Automatic Item Selection Procedure [*aisp*], Principal Component Analysis (PCA), and exploratory and confirmatory Factor Analysis [EFA and CFA] using R’s [71] packages factoextra, NbClust, mokken, psych, and lavaan. These seem to suggest that a one‐factor model best fits the human raters, while a two‐factor model might fit the GPT‐4o data slightly better than the one‐factor model, but, combined with the fact that we only have five dimensions and one latent with only two problematic indicators, we decided to fit a one‐factor model to these ratings as well. Nevertheless, fitting a one‐factor model to all three sets of ratings (using lavaan’s multiple groups) clearly shows that both the factor loadings and the latent means are significantly different but still numerically similar enough, prompting us to estimate this latent Creativity score separately for the Human, GPT‐4o, and Guided‐GPT‐4o raters.

Third, we tested the effects of various predictors on this Creativity score using mixed effects Beta regressions (as these scores are, by definition, bounded) with the item and rater IDs as crossed random effects using R’s [71] glmmTMB package, and their results were plotted using sjPlot.

Finally, we assessed the correspondence between Human and both GPT‐4o and Guided‐GPT‐4o ratings across the five rated subscales and the overall Creativity score using Pearson correlations. Differences in correlation strength were tested using Fisher's r‐to‐z transformations.

## Author Contributions

SR: Conceptualization; data curation; formal analysis; writing – original draft. CAM: Data curation; formal analysis; writing – original draft. PA: Conceptualization; writing – review and editing. OP: Formal analysis; writing – review and editing. MPa: Conceptualization; data curation; writing – review and editing. MPe: Conceptualization; writing – review and editing. DD: Formal analysis; writing – review and editing. ARF: Conceptualization; funding acquisition; project administration; resources; supervision; writing – review and editing. XCC: Conceptualization; data curation; funding acquisition; project administration; resources; supervision; writing – review and editing.

## Funding

This project received funding from a grant PID2023‐151083NA‐I00 funded by MICIU/AEI/10.13039/501100011033 and by ERDF/EU, grant PID2021‐127130NB‐100 funded by MICIU/AEI/10.13039/501100011033 and by ERDF/EU, the project Cátedra ENIA UAB‐Cruïlla (TSI‐100929‐2023‐2), the project Retech (SDC007/25/000029, and SDC007/25/000005), and the European Researchers’ Night and Researchers at Schools 2024–2025 (no. 101162558) by the European Performing Science Program (EPSP). SR has been awarded a Maria Capdevila Research Fellowship from the Computer Vision Center. CAM was supported by the Spanish Government with a predoctoral FPI fellowship (MCI22020), and ARF was supported by the FIAS fellowship Program, co‐funded by the European Commission, Marie‐Skłodowska‐Curie Actions—COFUND Program, Grant no 945408. DD acknowledges grant number PID2022‐138501NB‐I00 funded by MICIU/AEI/10.13039/501100011033 (Spain) and by ERDF/EU.

## Conflicts of Interest

The authors declare no conflict of interest.

## Supporting information




**Supporting File 1**: advs74695‐sup‐0001‐SuppMat.docx.

**Supporting File 2**: advs74695‐sup‐0002‐Table_S21.pdf.

## Data Availability

The data that support the findings of this study are available from the corresponding author upon reasonable request.
