# Supplementary material for: Stable Diffusion Models Reveal a Persisting Human–AI Gap in Visual Creativity
Source: Adv Sci (Weinh). 2026 Mar 24;13(27):e24142. doi: 10.1002/advs.202524142 (PMC13170252; doi:10.1002/advs.202524142)
Supplement: Supplementary file 1 — Supporting File 1: advs74695‐sup‐0001‐SuppMat.docx. [file ADVS-13-e24142-s001.docx]

Supplementary Materials for

**Stable diffusion models reveal a persisting human–AI gap in**

**visual creativity**

S. Rondini^1,2†^, C. Alvarez-Martin^1,3†^, P. Angermair-Barkai^4,5^, O. Penacchio^2,6^, M. Paz^2^, M. Pelowski^4,5^, D. Dediu^7,8,9^, A. Rodriguez-Fornells^1,3,9,10*‡^, X. Cerda-Company^2,6*‡^

^1^ Cognition and Brain Plasticity Unit, Bellvitge Biomedical Research Institute, L'Hospitalet de Llobregat, Spain

^2^ Bridging Research in AI and Neuroscience, Computer Vision Center, Bellaterra, Spain

^3^ Department of Cognition, Development and Educational Psychology, University of Barcelona, Barcelona, Spain

^4^ [Vienna Cognitive Science Hub](https://ufind.univie.ac.at/en/pvz_sub.html?id=220), Vienna, Austria

^5^ Faculty of Psychology, University of Vienna, Vienna, Austria

^6^ Computer Science Department, Universitat Autonoma de Barcelona, Bellaterra, Spain.

^7^ University of Barcelona Institute for Complex Systems (UBICS), Barcelona, Spain

^8^ Department of Catalan Philology and General Linguistics, University of Barcelona, Barcelona, Spain

^9^ Catalan Institution for Research and Advanced Studies (ICREA), Barcelona, Spain

^10^ Aix-Marseille University, Iméra, Marseille, F-13000, France

* Corresponding authors

^†^ These authors contributed equally

^‡^ Equal senior contribution

**Supplementary Text**

**Section S1. Creativity dimensions selection**

The “Vividness” criterion refers to the richness and realism of the physical characteristics of the generated image, in its visualisation and execution. In the TCIA, it essentially mirrors the “Elaboration” criterion of classical verbal DT tasks. The “Originality” criterion refers to the novelty and innovativeness of the content of the generated image, and how distant the underlying idea is from the norm. It constitutes one of Guilford’s [*1*] four divergent thinking parameters, and was described as ‘‘the ability to produce ideas that are generally not produced, or ideas that are totally new and unique.’’ [*2*]. “Liking” was included to gauge a first holistic impression of the creative image, the most basic possible measure for the viewer’s initial appraisal, whether positive or negative. The “Aesthetics” criterion reflects one’s ability to appreciate and respond to beauty, aesthetical reactions emerging from the appraisal of complex creative work, eliciting strong psychological states of curiosity and emotional intensity [*3*, *4*]. Lastly, the “Curiosity” criterion aims to capture the extent to which an artwork succeeds in drawing a viewer’s attention and interest towards itself, it be through its form or content, and was included to incorporate recent findings linking it to both aesthetic and creative experiences [*3*].

**Section S2. Fine-tuning information and image generation parameters.**

This study employed Stable Diffusion XL Base 1.0 [*5*], which was fine-tuned on a dataset of 1,020 TCIA drawings (660 from in-lab trials from Phase I, 360 from an art event with 30 artists) using Kohya v22.6.0 on PyTorch 2.1.2 (CUDA 11.8) with NVIDIA RTX 3090 infrastructure. The Low-Rank Adaptation (LoRA) fine-tuning process utilized a batch size of 4 across 5 epochs, with an AdaFactor optimizer, a learning rate of 0.003, and a resolution of 1024×1024. Network parameters were set to a dimension 16 and an alpha 4.

Image generation in the GenAI group was carried out through Automatic1111's interface with LoRA weight of 0.8, employing DPM++ 2M sampling with automated scheduling (30 steps) and ControlNet v1.1.448 integration. For each generation, a stimulus from the TCIA was used as the input for ControlNet, using Canny edge detection (thresholds: 100/200).

Two distinct sets of GenAI images were produced using tailored parameter configurations to explore variation in aesthetic and compositional outcomes. The HI-GenAI set was generated using a CFG (classifier-free guidance) scale ranging from 5.5 to 8, control weight values between 1.2 and 1.4, and control steps ranging from 0 to 0.4. The SG-GenAI set employed a CFG scale between 7 and 8, control weights from 1.0 to 1.4, and control steps between 0 and 0.2–0.4. Finally, a total of 452 images were generated: 230 with **[idea]** as prompt, and 222 without.

For the Diffusion groups, the original TCIA patterns were re-imposed onto the generated drawings to ensure visual consistency with the human results.

**Section S3. Adapted TCIA scoring criteria and respective questions for the image rating task for Phases III and IV.**

1. **Liking**: “How much do you personally like the drawing?”
2. **Vividness**: “How rich and detailed is the imagery in the drawing?”
3. **Originality**: “How novel, creative, unconventional is the drawing?”
4. **Aesthetics**: “To what extent does the drawing evoke aesthetic feelings (e.g. beauty, fascination, awe)?”
5. **Curiosity & Interest**: “To what extent does the drawing elicit your curiosity or interest?

**Section S4. Turing Test.**

Although creativity raters were blind to the origin of the images and were not informed that any drawings could be generated by an artificial model, we conducted an additional Turing-style classification task to assess whether participants could nevertheless infer image authorship from visual characteristics alone. This control analysis was motivated by the possibility that even without explicit instruction, raters might implicitly suspect multiple image sources, potentially biasing creativity judgments.

**Methods**

Fifty human participants (26 Male, 24 Female; mean age = 32.7 ± 9.9 y.o.) completed the Turing-style task through the Socrative platform. Unlike the creativity raters, these participants were explicitly informed that drawings could originate from either humans or GenAI. The task consisted of a two-alternative forced-choice paradigm, in which participants classified each drawing as either human- or AI-generated. After each judgment, participants rated their confidence on a 5-point Likert scale.

A total of 48 drawings were randomly selected from the original dataset, with sampling constraint to ensure a balanced representation of low and high creativity scores across all four experimental categories (Human Visual Artist, Human Non-Visual Artists, HI-GenAI, and SG-GenAI). A Kruskall-Wallis test confirmed that creativity score distributions did not differ significantly across categories (H = 2.79; p = .4248).

We defined successful performance in the Turing-style task as an average participant accuracy below 0.70 for AI-generated drawings, corresponding to more than 30% of participants misclassifying their origin [6].

**Results**

Our architecture successfully passed the Turing-style classification task (mean accuracy = 0.65). Moreover, the percentages of confusion between AI and human generated images were not significantly different (accuracy for human-generated images = 0.68, accuracy for GenAI-generated images = 0.62, χ(1)=2.75, p = .097).

In summary, these results make it unlikely that, even if naïve raters had formed implicit inferences about the drawings’ origin, their limited accuracy would have produced a systematic bias favoring either category of drawings.


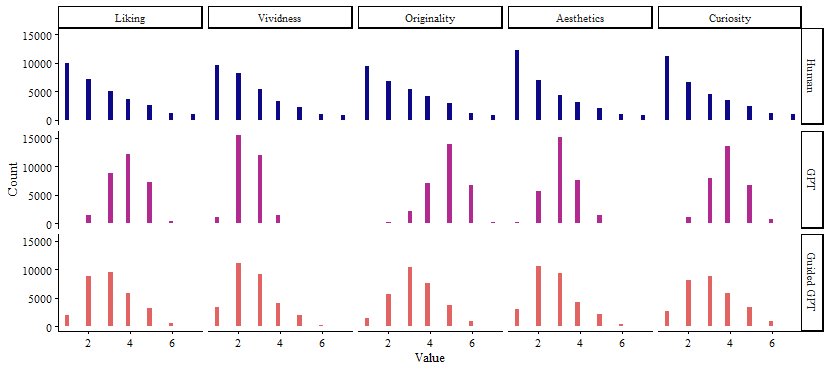


**Fig. S1.** Histograms comparing the scores on the five creativity dimensions between GenAI and human raters across categories.


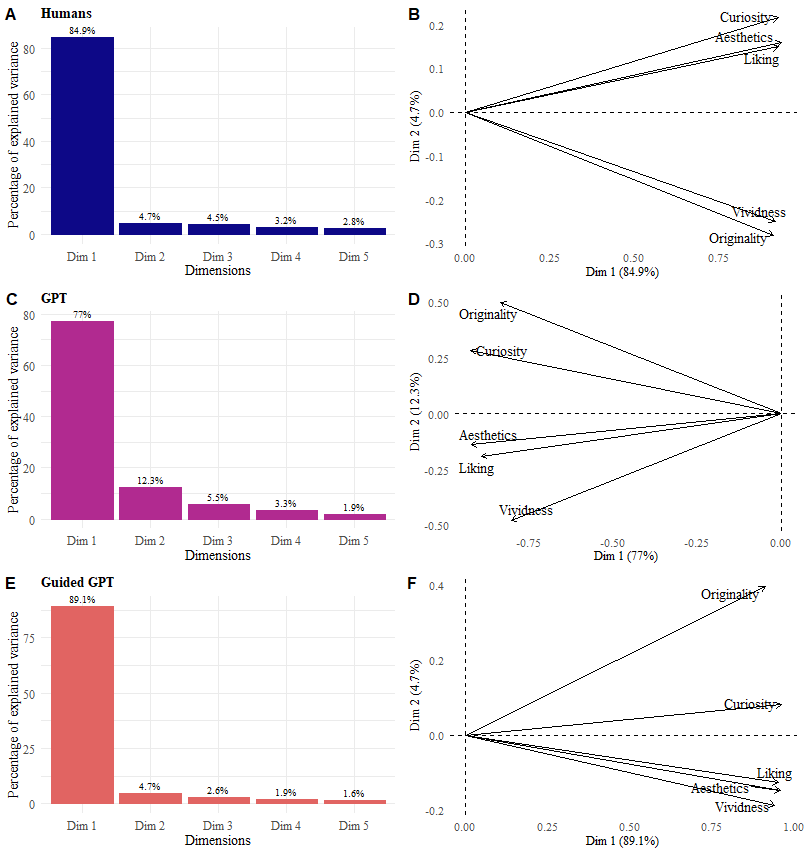


**Fig. S2.** Principal component analysis (PCA) of creativity ratings. For the human ratings (A), the first principal component explains 84.9% of the variance, followed by minor contributions from subsequent components. For the GPT-4o ratings (C), the first principal component explains 78.3% of the variance, and the 89.1% for the Guided-GPT-4o (E). Panels B, D and F show the correlation circle plots of the first two principal components.

**
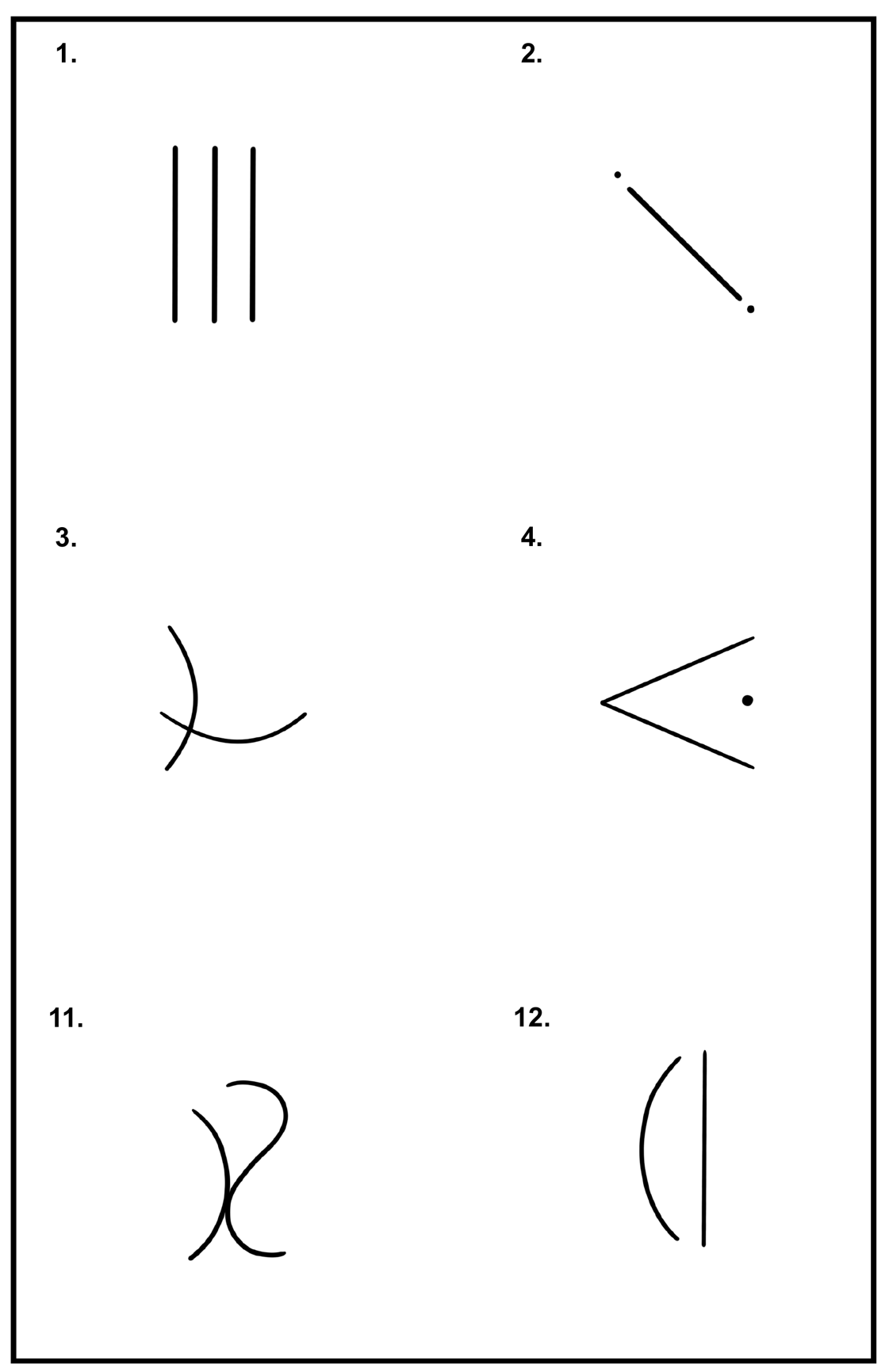
**

**Fig. S3.** Original TCIA Stimuli. Numbers on the top left-hand corner refer to the stimuli labels as they appear in the models.

**
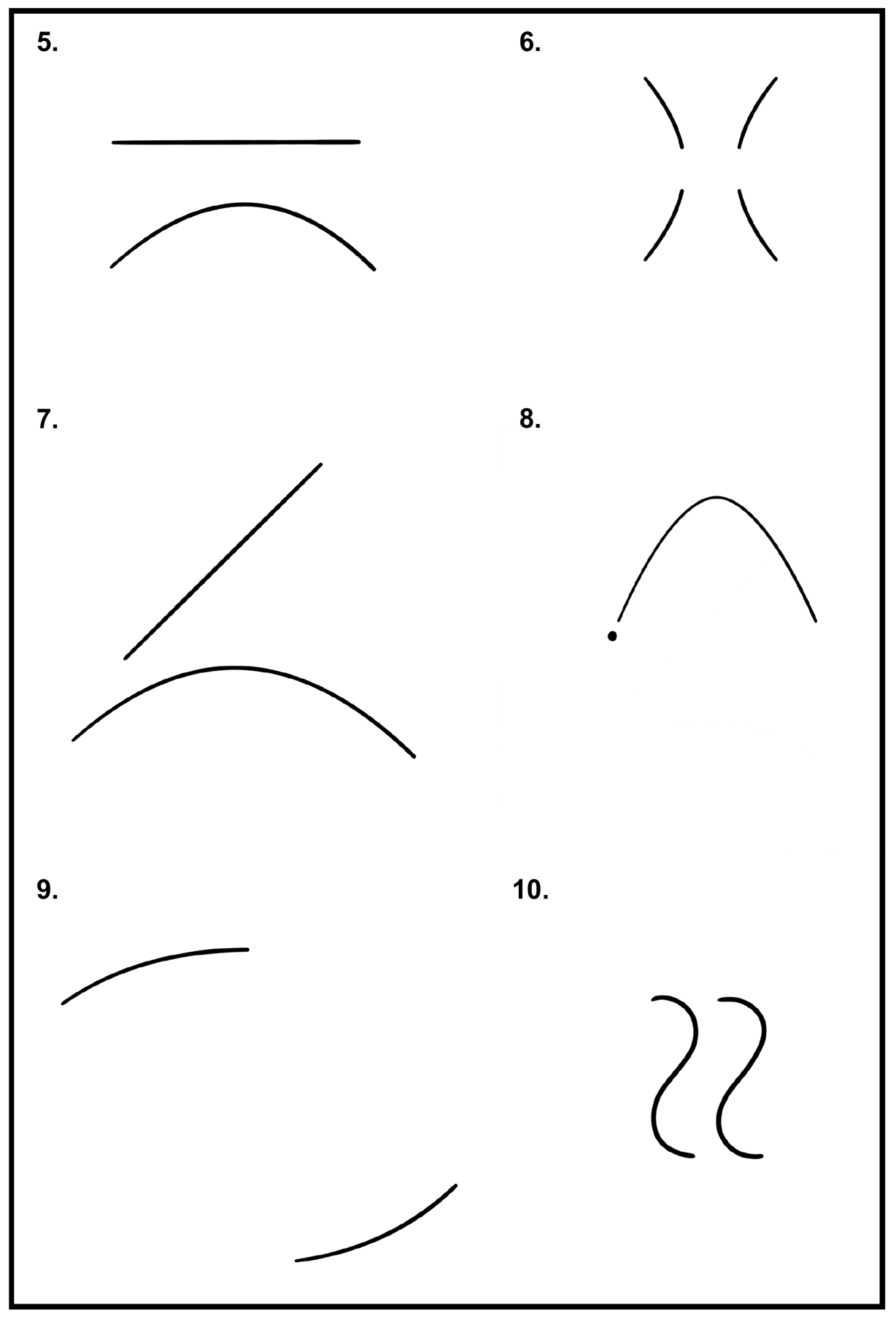
**

**Fig. S4.** Newly created stimuli, mimicking the original TCIA format. Numbers on the top left-hand corner refer to the stimuli labels as they appear in the models.

| Fixed Effects |  |  |  |  |  |
| --- | --- | --- | --- | --- | --- |
|  | β | SE | 95% CI | t | p value |
|  |  |  |  |  |  |
| Intercept | 3.4 | 0.02 | 3.36 - 3.44 | 168.31 | < .001 |
| Type of drawer (GenAI) | -0.45 | 0.03 | -0.51 - -0.4 | -15.1 | < .001 |
|  |  |  |  |  |  |
| Random effects |  |  |  |  |  |
|  |  |  |  | Variance | SD |
| Image (Intercept) |  |  |  | 0.177 | 0.421 |
|  |  |  |  |  |  |
| Model fit |  |  |  |  |  |
|  |  |  |  | Marginal | Conditional |
| R2 |  |  |  | 0.03 | 0.14 |

**Table S1.** Model summary: effect of image category on overall Creativity score in human raters.

| Fixed Effects |  |  |  |  |  |
| --- | --- | --- | --- | --- | --- |
|  | β | SE | 95% CI | t | p value |
|  |  |  |  |  |  |
| Intercept | 3.57 | 0.03 | 3.52 - 3.62 | 138.14 | < .001 |
| Category (Visual Artists) |  |  |  |  |  |
| Non-Artists | -0.35 | 0.04 | -0.43 - -0.28 | -9.48 | < .001 |
| Human-Inspired GenAI | -0.43 | 0.04 | -0.5 - -0.35 | -11.1 | < .001 |
| Self-Guided GenAI | -0.83 | 0.04 | -0.9 - -0.75 | -21.21 | < .001 |
|  |  |  |  |  |  |
| Random effects |  |  |  |  |  |
|  |  |  |  | Variance | SD |
| Image (Intercept) |  |  |  | 0.142 | 0.377 |
|  |  |  |  |  |  |
| Model fit |  |  |  |  |  |
|  |  |  |  | Marginal | Conditional |
| R2 |  |  |  | 0.05 | 0.14 |

**Table S2.** Model summary: effect of drawer type on overall Creativity score for human raters.

| Fixed Effects |  |  |  |  |  |
| --- | --- | --- | --- | --- | --- |
|  | β | SE | 95% CI | t | p value |
|  |  |  |  |  |  |
| Intercept | 3.14 | 0.03 | 3.07 - 3.2 | 95.42 | < .001 |
| Category (Visual Artists) |  |  |  |  |  |
| Non-Artists | -0.41 | 0.05 | -0.5 - -0.31 | -8.59 | < .001 |
| Human-Inspired GenAI | -0.59 | 0.05 | -0.69 - -0.49 | -11.99 | < .001 |
| Self-Guided GenAI | -1.07 | 0.05 | -1.17 - -0.98 | -21.63 | < .001 |
|  |  |  |  |  |  |
| Random effects |  |  |  |  |  |
|  |  |  |  | Variance | SD |
| Image (Intercept) |  |  |  | 0.228 | 0.478 |
|  |  |  |  |  |  |
| Model fit |  |  |  |  |  |
|  |  |  |  | Marginal | Conditional |
| R2 |  |  |  | 0.05 | 0.14 |

**Table S3.** Model summary: effect of image category on Liking in human raters.

| Fixed Effects |  |  |  |  |  |
| --- | --- | --- | --- | --- | --- |
|  | β | SE | 95% CI | t | p value |
|  |  |  |  |  |  |
| Intercept | 3.08 | 0.04 | 3.01 - 3.15 | 87.57 | < .001 |
| Category (Visual Artists) |  |  |  |  |  |
| Non-Artists | -0.45 | 0.05 | -0.55 - -0.35 | -8.83 | < .001 |
| Human Inspired GenAI | -0.59 | 0.05 | -0.69 - -0.49 | -11.27 | < .001 |
| Self-Guided GenAI | -1.1 | 0.05 | -1.2 - -0.99 | -20.71 | < .001 |
|  |  |  |  |  |  |
| Random effects |  |  |  |  |  |
|  |  |  |  | Variance | SD |
| Image (Intercept) |  |  |  | 0.282 | 0.531 |
|  |  |  |  |  |  |
| Model fit |  |  |  |  |  |
|  |  |  |  | Marginal | Conditional |
| R2 |  |  |  | 0.06 | 0.17 |

**Table S4.** Model summary: effect of image category on Vividness in human raters.

| Fixed Effects |  |  |  |  |  |
| --- | --- | --- | --- | --- | --- |
|  | β | SE | 95% CI | t | p value |
|  |  |  |  |  |  |
| Intercept | 3.16 | 0.03 | 3.1 - 3.23 | 98.88 | < .001 |
| Category (Visual Artists) |  |  |  |  |  |
| Non-Artists | -0.44 | 0.05 | -0.53 - -0.35 | -9.5 | < .001 |
| Human Inspired GenAI | -0.5 | 0.05 | -0.6 - -0.41 | -10.55 | < .001 |
| Self-Guided GenAI | -0.95 | 0.05 | -1.05 - -0.86 | -19.71 | < .001 |
|  |  |  |  |  |  |
| Random effects |  |  |  |  |  |
|  |  |  |  | Variance | SD |
| Image (Intercept) |  |  |  | 0.212 | 0.461 |
|  |  |  |  |  |  |
| Model fit |  |  |  |  |  |
|  |  |  |  | Marginal | Conditional |
| R2 |  |  |  | 0.04 | 0.12 |

**Table S5.** Model summary: effect of image category on Originality in human raters.

| Fixed Effects |  |  |  |  |  |
| --- | --- | --- | --- | --- | --- |
|  | β | SE | 95% CI | t | p value |
|  |  |  |  |  |  |
| Intercept | 2.78 | 0.03 | 2.72 - 2.83 | 96.59 | < .001 |
| Category (Visual Artists) |  |  |  |  |  |
| Non-Artists | -0.36 | 0.04 | -0.44 - -0.28 | -8.7 | < .001 |
| Human-Inspired GenAI | -0.41 | 0.04 | -0.5 - -0.33 | -9.62 | < .001 |
| Self-Guided GenAI | -0.84 | 0.04 | -0.93 - -0.76 | -19.41 | < .001 |
|  |  |  |  |  |  |
| Random effects |  |  |  |  |  |
|  |  |  |  | Variance | SD |
| Image (Intercept) |  |  |  | 0.159 | 0.398 |
|  |  |  |  |  |  |
| Model fit |  |  |  |  |  |
|  |  |  |  | Marginal | Conditional |
| R2 |  |  |  | 0.03 | 0.1 |

**Table S6.** Model summary: effect of image category on Aesthetics in human raters.

| Fixed Effects |  |  |  |  |  |
| --- | --- | --- | --- | --- | --- |
|  | β | SE | 95% CI | t | p value |
|  |  |  |  |  |  |
| Intercept | 2.98 | 0.03 | 2.92 - 3.04 | 98.27 | < .001 |
| Category (Visual Artists) |  |  |  |  |  |
| Non-Artists | -0.43 | 0.04 | -0.51 - -0.34 | -9.84 | < .001 |
| Human-Inspired GenAI | -0.44 | 0.05 | -0.53 - -0.35 | -9.63 | < .001 |
| Self-Guided GenAI | -0.9 | 0.05 | -0.99 - -0.82 | -19.78 | < .001 |
|  |  |  |  |  |  |
| Random effects |  |  |  |  |  |
|  |  |  |  | Variance | SD |
| Image (Intercept) |  |  |  | 0.179 | 0.423 |
|  |  |  |  |  |  |
| Model fit |  |  |  |  |  |
|  |  |  |  | Marginal | Conditional |
| R2 |  |  |  | 0.04 | 0.1 |

**Table S7.** Model summary: effect of image category on Curiosity in human raters.

| Fixed Effects |  |  |  |  |  |
| --- | --- | --- | --- | --- | --- |
|  | β | SE | 95% CI | t | p value |
|  |  |  |  |  |  |
| Intercept | 3.23 | 0.03 | 3.16 - 3.29 | 100.73 | < .001 |
| Category (Visual Artists) |  |  |  |  |  |
| Non-Artists | -0.01 | 0.05 | -0.1 - 0.08 | -0.28 | 0.781 |
| Human Inspired GenAI | 0.1 | 0.05 | 0.01 - 0.2 | 2.13 | 0.034 |
| Self-Guided GenAI | -0.22 | 0.05 | -0.32 - -0.13 | -4.56 | < .001 |
|  |  |  |  |  |  |
| Random effects |  |  |  |  |  |
|  |  |  |  | Variance | SD |
| Image (Intercept) |  |  |  | 0.286 | 0.535 |
|  |  |  |  |  |  |
| Model fit |  |  |  |  |  |
|  |  |  |  | Marginal | Conditional |
| R2 |  |  |  | 0.03 | 0.7 |

**Table S8.** Model summary: effect of image category on Creativity score according to GPT-4o ratings.

| Fixed Effects |  |  |  |  |  |
| --- | --- | --- | --- | --- | --- |
|  | β | SE | 95% CI | t | p value |
|  |  |  |  |  |  |
| Intercept | 3.63 | 0.04 | 3.56 - 3.7 | 98.75 | < .001 |
| Category (Visual Artists) |  |  |  |  |  |
| Non-Artists | -0.23 | 0.05 | -0.34 - -0.13 | -4.42 | < .001 |
| Human Inspired GenAI | -0.56 | 0.05 | -0.67 - -0.46 | -10.27 | < .001 |
| Self-Guided GenAI | -1.09 | 0.06 | -1.2 - -0.98 | -19.61 | < .001 |
|  |  |  |  |  |  |
| Random effects |  |  |  |  |  |
|  |  |  |  | Variance | SD |
| Image (Intercept) |  |  |  | 0.37 | 0.608 |
|  |  |  |  |  |  |
| Model fit |  |  |  |  |  |
|  |  |  |  | Marginal | Conditional |
| R2 |  |  |  | 0.18 | 0.59 |

**Table S9.** Model summary: effect of image category on Creativity score according to Guided-GPT-4o ratings.

| Fixed effects |  |  |  |  |  |
| --- | --- | --- | --- | --- | --- |
|  | β | SE | 95% CI | t | p value |
|  |  |  |  |  |  |
| Intercept | 3.98 | 0.04 | 3.9 - 4.06 | 99.25 | < .001 |
| Category (Visual Artists) |  |  |  |  |  |
| Non-Artists | 0 | 0.06 | -0.11 - 0.12 | 0.08 | 0.936 |
| Human Inspired GenAI | -0.01 | 0.06 | -0.13 - 0.1 | -0.22 | 0.83 |
| Self-Guided GenAI | -0.48 | 0.06 | -0.6 - -0.36 | -7.93 | < .001 |
|  |  |  |  |  |  |
| Random effects |  |  |  |  |  |
|  |  |  |  | Variance | SD |
| Image (Intercept) |  |  |  | 0.445 | 0.667 |
|  |  |  |  |  |  |
| Model fit |  |  |  |  |  |
|  |  |  |  | Marginal | Conditional |
| R2 |  |  |  | 0.05 | 0.62 |

## Table S10. Model summary: effect of image category on Liking according to GPT-4o ratings.

| Fixed Effects |  |  |  |  |  |
| --- | --- | --- | --- | --- | --- |
|  | β | SE | 95% CI | t | p value |
|  |  |  |  |  |  |
| Intercept | 2.56 | 0.03 | 2.5 - 2.62 | 84.92 | <.001 |
| Category (Visual Artists) |  |  |  |  |  |
| Non-Artists | 0.03 | 0.04 | -0.06 - 0.11 | 0.66 | 0.509 |
| Human Inspired GenAI | -0.08 | 0.05 | -0.16 - 0.01 | -1.7 | 0.089 |
| Self-Guided GenAI | -0.38 | 0.05 | -0.46 - -0.29 | -8.27 | <.001 |
|  |  |  |  |  |  |
| Random effects |  |  |  |  |  |
|  |  |  |  | Variance | SD |
| Image (Intercept) |  |  |  | 0.252 | 0.502 |
|  |  |  |  |  |  |
| Model fit |  |  |  |  |  |
|  |  |  |  | Marginal | Conditional |
| R2 |  |  |  | 0.06 | 0.64 |

## Table S11. Model Summary: effect of image category on Vividness according to GPT-4o ratings.

| Fixed Effects |  |  |  |  |  |
| --- | --- | --- | --- | --- | --- |
|  | β | SE | 95% CI | t | p value |
|  |  |  |  |  |  |
| Intercept | 4.78 | 0.04 | 4.7 - 4.87 | 112.19 | <.001 |
| Category (Visual Artists) |  |  |  |  |  |
| Non-Artists | -0.02 | 0.06 | -0.14 - 0.1 | -0.29 | 0.772 |
| Human Inspired GenAI | 0.29 | 0.06 | 0.16 - 0.41 | 4.54 | <.001 |
| Self-Guided GenAI | 0.01 | 0.06 | -0.11 - 0.14 | 0.18 | 0.858 |
|  |  |  |  |  |  |
| Random effects |  |  |  |  |  |
|  |  |  |  | Variance | SD |
| Image (Intercept) |  |  |  | 0.506 | 0.711 |
|  |  |  |  |  |  |
| Model fit |  |  |  |  |  |
|  |  |  |  | Marginal | Conditional |
| R2 |  |  |  | 0.02 | 0.67 |

## Table S12. Model Summary: effect of image category on Originality according to GPT-4o ratings.

| Fixed Effects |  |  |  |  |  |
| --- | --- | --- | --- | --- | --- |
|  | β | SE | 95% CI | t | p value |
|  |  |  |  |  |  |
| Intercept | 3.19 | 0.04 | 3.11 - 3.26 | 83.47 | <.001 |
| Category (Visual Artists) |  |  |  |  |  |
| Non-Artists | -0.05 | 0.05 | -0.16 - 0.06 | -0.87 | 0.382 |
| Human Inspired GenAI | 0.12 | 0.06 | 0 - 0.23 | 2.04 | 0.042 |
| Self-Guided GenAI | -0.27 | 0.06 | -0.38 - -0.15 | -4.64 | <.001 |
|  |  |  |  |  |  |
| Random effects |  |  |  |  |  |
|  |  |  |  | Variance | SD |
| Image (Intercept) |  |  |  | 0.405 | 0.637 |
|  |  |  |  |  |  |
| Model fit |  |  |  |  |  |
|  |  |  |  | Marginal | Conditional |
| R2 |  |  |  | 0.03 | 0.64 |

**Table S13.** Model Summary: effect of image category on Aesthetics according to GPT-4o ratings.

| Fixed Effects |  |  |  |  |  |
| --- | --- | --- | --- | --- | --- |
|  | β | SE | 95% CI | t | p value |
|  |  |  |  |  |  |
| Intercept | 3.93 | 0.04 | 3.85 - 4.01 | 95.4 | <.001 |
| Category (Visual Artists) |  |  |  |  |  |
| Non-Artists | -0.01 | 0.06 | -0.12 - 0.11 | -0.14 | 0.888 |
| Human Inspired GenAI | 0.21 | 0.06 | 0.09 - 0.33 | 3.38 | 0.001 |
| Self-Guided GenAI | -0.14 | 0.06 | -0.26 - -0.02 | -2.3 | 0.022 |
|  |  |  |  |  |  |
| Random effects |  |  |  |  |  |
|  |  |  |  | Variance | SD |
| Image (Intercept) |  |  |  | 0.47 | 0.686 |
|  |  |  |  |  |  |
| Model fit |  |  |  |  |  |
|  |  |  |  | Marginal | Conditional |
| R2 |  |  |  | 0.2 | 0.65 |

**Table S14.** Model Summary: effect of image category on Curiosity according to GPT-4o ratings.

| Fixed effects |  |  |  |  |  |
| --- | --- | --- | --- | --- | --- |
|  | β | SE | 95% CI | t | p value |
|  |  |  |  |  |  |
| Intercept | 3.59 | 0.04 | 3.51 - 3.67 | 85.27 | < .001 |
| Category (Visual Artists) |  |  |  |  |  |
| Non-Artists | -0.27 | 0.06 | -0.38 - -0.15 | -4.39 | < .001 |
| Human Inspired GenAI | -0.74 | 0.06 | -0.86 - -0.61 | -11.73 | < .001 |
| Self-Guided GenAI | -1.36 | 0.06 | -1.48 - -1.24 | -21.42 | < .001 |
|  |  |  |  |  |  |
| Random effects |  |  |  |  |  |
|  |  |  |  | Variance | SD |
| Image (Intercept) |  |  |  | 0.48 | 0.693 |
|  |  |  |  |  |  |
| Model fit |  |  |  |  |  |
|  |  |  |  | Marginal | Conditional |
| R2 |  |  |  | 0.19 | 0.54 |

## Table S15. Model summary: effect of image category on Liking according to Guided-GPT-4o ratings.

| Fixed Effects |  |  |  |  |  |
| --- | --- | --- | --- | --- | --- |
|  | β | SE | 95% CI | t | p value |
|  |  |  |  |  |  |
| Intercept | 3.2 | 0.04 | 3.12 - 3.29 | 72.74 | <.001 |
| Category (Visual Artists) |  |  |  |  |  |
| Non-Artists | -0.26 | 0.06 | -0.39 - -0.14 | -4.14 | <.001 |
| Human Inspired GenAI | -0.67 | 0.07 | -0.8 - -0.54 | -10.2 | <.001 |
| Self-Guided GenAI | -1.23 | 0.07 | -1.36 - -1.1 | -18.47 | <.001 |
|  |  |  |  |  |  |
| Random effects |  |  |  |  |  |
|  |  |  |  | Variance | SD |
| Image (Intercept) |  |  |  | 0.533 | 0.73 |
|  |  |  |  |  |  |
| Model fit |  |  |  |  |  |
|  |  |  |  | Marginal | Conditional |
| R2 |  |  |  | 0.17 | 0.61 |

## Table S16. Model Summary: effect of image category on Vividness according to Guided-GPT-4o ratings.

| Fixed Effects |  |  |  |  |  |
| --- | --- | --- | --- | --- | --- |
|  | β | SE | 95% CI | t | p value |
|  |  |  |  |  |  |
| Intercept | 3.78 | 0.04 | 3.7 - 3.87 | 86.65 | <.001 |
| Category (Visual Artists) |  |  |  |  |  |
| Non-Artists | -0.3 | 0.06 | -0.42 - -0.17 | -4.74 | <.001 |
| Human Inspired GenAI | -0.57 | 0.07 | -0.7 - -0.44 | -8.78 | <.001 |
| Self-Guided GenAI | -1.17 | 0.07 | -1.3 - -1.04 | -17.81 | <.001 |
|  |  |  |  |  |  |
| Random effects |  |  |  |  |  |
|  |  |  |  | Variance | SD |
| Image (Intercept) |  |  |  | 0.518 | 0.72 |
|  |  |  |  |  |  |
| Model fit |  |  |  |  |  |
|  |  |  |  | Marginal | Conditional |
| R2 |  |  |  | 0.13 | 0.52 |

## Table S17. Model Summary: effect of image category on Originality according to Guided-GPT-4o ratings.

| Fixed Effects |  |  |  |  |  |
| --- | --- | --- | --- | --- | --- |
|  | β | SE | 95% CI | t | p value |
|  |  |  |  |  |  |
| Intercept | 3.2 | 0.04 | 3.12 - 3.29 | 75.13 | <.001 |
| Category (Visual Artists) |  |  |  |  |  |
| Non-Artists | -0.24 | 0.06 | -0.36 - -0.12 | -3.86 | <.001 |
| Human Inspired GenAI | -0.54 | 0.06 | -0.66 - -0.41 | -8.43 | <.001 |
| Self-Guided GenAI | -1.13 | 0.06 | -1.25 - -1 | -17.5 | <.001 |
|  |  |  |  |  |  |
| Random effects |  |  |  |  |  |
|  |  |  |  | Variance | SD |
| Image (Intercept) |  |  |  | 0.495 | 0.704 |
|  |  |  |  |  |  |
| Model fit |  |  |  |  |  |
|  |  |  |  | Marginal | Conditional |
| R2 |  |  |  | 0.14 | 0.53 |

**Table S18.** Model Summary: effect of image category on Aesthetics according to Guided-GPT-4o ratings.

| Fixed Effects |  |  |  |  |  |
| --- | --- | --- | --- | --- | --- |
|  | β | SE | 95% CI | t | p value |
|  |  |  |  |  |  |
| Intercept | 3.66 | 0.05 | 3.56 - 3.75 | 75.65 | <.001 |
| Category (Visual Artists) |  |  |  |  |  |
| Non-Artists | -0.33 | 0.07 | -0.46 - -0.19 | -4.69 | <.001 |
| Human Inspired GenAI | -0.76 | 0.07 | -0.9 - -0.62 | -10.57 | <.001 |
| Self-Guided GenAI | -1.44 | 0.07 | -1.58 - -1.3 | -19.76 | <.001 |
|  |  |  |  |  |  |
| Random effects |  |  |  |  |  |
|  |  |  |  | Variance | SD |
| Image (Intercept) |  |  |  | 0.639 | 0.799 |
|  |  |  |  |  |  |
| Model fit |  |  |  |  |  |
|  |  |  |  | Marginal | Conditional |
| R2 |  |  |  | 0.18 | 0.58 |

**Table S19.** Model Summary: effect of image category on Curiosity according to Guided-GPT-4o ratings.

| Fixed Effects |  |  |  |  |  |
| --- | --- | --- | --- | --- | --- |
|  | β | SE | 95% CI | t | p value |
|  |  |  |  |  |  |
| (Intercept) | 3.57 | 0.03 | 3.52 - 3.63 | 123.65 | < .001 |
| Rater (Human) |  |  |  |  |  |
| GPT-4o | -0.35 | 0.01 | -0.37 - -0.32 | -27.2 | < .001 |
| Guided-GPT-4o | 0.06 | 0.01 | 0.03 - 0.08 | 4.37 | < .001 |
| Category (Visual Artists) |  |  |  |  |  |
| Non-Artists | -0.35 | 0.04 | -0.43 - -0.27 | -8.5 | < .001 |
| Human-Inspired GenAI | -0.43 | 0.04 | -0.51 - -0.35 | -9.96 | < .001 |
| Self-Guided GenAI | -0.83 | 0.04 | -0.91 - -0.74 | -19 | < .001 |
| Category (Visual Artists) x Human Raters |  |  |  |  |  |
| Non-Artists – GPT-4o Rater | 0.34 | 0.02 | 0.3 - 0.38 | 18.52 | < .001 |
| Non-Artists – Guided-GPT-4o Rater | 0.12 | 0.02 | 0.08 - 0.16 | 6.5 | < .001 |
| Human-Inspired GenAI – GPT-4o Rater | 0.53 | 0.02 | 0.49 - 0.57 | 27.83 | < .001 |
| Human-Inspired GenAI – Guided-GPT-4o Rater | -0.13 | 0.02 | -0.17 - -0.1 | -7.01 | < .001 |
| Self-Guided GenAI – GPT-4o Rater | 0.61 | 0.02 | 0.57 - 0.65 | 31.52 | < .001 |
| Self-Guided GenAI – Guided-GPT-4o Rater | -0.26 | 0.02 | -0.3 - -0.22 | -13.41 | < .001 |
|  |  |  |  |  |  |
| Random effects |  |  |  |  |  |
|  |  |  |  | Variance | SD |
| Image (Intercept) |  |  |  | 0.214 | 0.462 |
|  |  |  |  |  |  |
| Model fit |  |  |  |  |  |
|  |  |  |  | Marginal | Conditional |
| R2 |  |  |  | 0.09 | 0.3 |

**Table S20.** Model Summary: Human vs GPT-4o ratings.

| Fixed Effects |  |  |  |  |  |
| --- | --- | --- | --- | --- | --- |
|  | β | SE | 95% CI | t | p value |
|  |  |  |  |  |  |
| (Intercept) | 3.87 | 0.03 | 3.81 - 3.92 | 133.78 | < .001 |
| Category (Visual Artists) |  |  |  |  |  |
| Non-Artists | -0.36 | 0.04 | -0.44 - -0.28 | -8.63 | < .001 |
| Human-Inspired GenAI | -0.43 | 0.04 | -0.52 - -0.35 | -10.04 | < .001 |
| Self-Guided GenAI | -0.85 | 0.04 | -0.94 - -0.77 | -19.55 | < .001 |
| AReA (Low) | -0.58 | 0.03 | -0.63 - -0.53 | -22.78 | < .001 |
| Category (Visual Artists) x AReA (High) |  |  |  |  |  |
| Non-Artists x AReA (Low) | 0 | 0.04 | -0.07 - 0.07 | 0.07 | 0.946 |
| Human-Inspired GenAI x AReA (Low) | 0 | 0.04 | -0.07 - 0.08 | 0.06 | 0.951 |
| Self-Guided GenAI x AreA (Low) | 0.04 | 0.04 | -0.03 - 0.12 | 1.08 | 0.28 |
|  |  |  |  |  |  |
| Random effects |  |  |  |  |  |
|  |  |  |  | Variance | SD |
| Image (Intercept) |  |  |  | 0.145 | 0.381 |
|  |  |  |  |  |  |
| Model fit |  |  |  |  |  |
|  |  |  |  | Marginal | Conditional |
| R2 |  |  |  | 0.1 | 0.19 |

**Table S22.** Model Summary: effect of High and Low AReA scores groups on each image category’s Creativity ratings.

**References**

1. Guilford, J. P. “Creativity: Yesterday, Today and Tomorrow.” Journal of Creative Behavior 1 (1967): 3–14.
2. Goff, K., and E. P. Torrance. Abbreviated Torrance Test for Adults. Washington, DC: American Psychological Association, 2012. <https://doi.org/10.1037/t12015-000>.
3. Kenett, Y. N., S. Humphries, and A. Chatterjee. “A Thirst for Knowledge: Grounding Curiosity, Creativity, and Aesthetics in Memory and Reward Neural Systems.” Creativity Research Journal 35 (2023): 412–426.
4. Vygotsky, L. S. The Psychology of Art. Cambridge, MA: MIT Press, 1974.
5. Stability AI. Stable Diffusion XL Base 1.0 [computer software]. 2023. [https://stability.ai](https://stability.ai/).
6. M. Mitchell. "The Turing Test and Our Shifting Conceptions of Intelligence." Science 385 (2024): eadq9356. <https://doi.org/10.1126/science.adq9356>
