# Supplementary material for: Stable Diffusion Models Reveal a Persisting Human–AI Gap in Visual Creativity
Source: Adv Sci (Weinh). 2026 Mar 24;13(27):e24142. doi: 10.1002/advs.202524142 (PMC13170252; doi:10.1002/advs.202524142)
Supplement: Supplementary file 2 — Supporting File 2: advs74695‐sup‐0002‐Table_S21.pdf. [file ADVS-13-e24142-s002.pdf]

Fixed effects

	Est/Beta	SE	95% CI
(Intercept)	3.44	0.1	3.25 - 3.62
Stimulus (1)			
Stimulus 2	0	0.14	-0.26 - 0.27
Stimulus 3	0.16	0.13	-0.1 - 0.43
Stimulus 4	0.14	0.14	-0.13 - 0.42
Stimulus 5	0.04	0.14	-0.23 - 0.31
Stimulus 6	0.23	0.14	-0.04 - 0.49
Stimulus 7	0.24	0.14	-0.03 - 0.5
Stimulus 8	0.04	0.14	-0.23 - 0.3
Stimulus 9	0.12	0.14	-0.14 - 0.39
Stimulus 10	0.24	0.13	-0.02 - 0.51
Stimulus 11	0.34	0.14	0.08 - 0.6
Stimulus 12	0.09	0.14	-0.18 - 0.36
Category (Visual Artists)			
Non Artists	-0.27	0.14	-0.54 - 0
Human Inspired GenAI	-0.55	0.14	-0.82 - -0.27
Self-Guided GenAI	-0.56	0.13	-0.81 - -0.31
Rater Type (Human)			
GPT	-0.3	0.03	-0.35 - -0.25
GuidedGPT	-0.03	0.03	-0.08 - 0.02
Stimulus (1) x Category (Visual artists)			
Stimulus 2:Non Artists	0.1	0.19	-0.29 - 0.48
Stimulus 3:Non Artists	-0.06	0.19	-0.43 - 0.32
Stimulus 4:Non Artists	-0.06	0.2	-0.45 - 0.32
Stimulus 5:Non Artists	-0.01	0.2	-0.41 - 0.38
Stimulus 6:Non Artists	-0.2	0.19	-0.58 - 0.17
Stimulus 7:Non Artists	-0.29	0.19	-0.67 - 0.08
Stimulus 8:Non Artists	-0.05	0.19	-0.43 - 0.33
Stimulus 9:Non Artists	-0.17	0.19	-0.55 - 0.21
Stimulus 10:Non Artists	-0.1	0.2	-0.49 - 0.28
Stimulus 11:Non Artists	-0.08	0.19	-0.46 - 0.29
Stimulus 12:Non Artists	-0.06	0.19	-0.44 - 0.32
Stimulus 2:Human Inspired GenAI	0.14	0.2	-0.25 - 0.54
Stimulus 3:Human Inspired GenAI	-0.3	0.22	-0.73 - 0.14
Stimulus 4:Human Inspired GenAI	0.02	0.19	-0.35 - 0.39
Stimulus 5:Human Inspired GenAI	0.48	0.19	0.11 - 0.86
Stimulus 6:Human Inspired GenAI	-0.09	0.19	-0.47 - 0.29
Stimulus 7:Human Inspired GenAI	0.17	0.2	-0.22 - 0.56
Stimulus 8:Human Inspired GenAI	0.28	0.19	-0.1 - 0.66
Stimulus 9:Human Inspired GenAI	0.21	0.2	-0.18 - 0.61
Stimulus 11:Human Inspired GenAI	0.16	0.2	-0.24 - 0.55
Stimulus 12:Human Inspired GenAI	0	0.25	-0.5 - 0.5
Stimulus 2:Self-Guided GenAI	-0.12	0.18	-0.48 - 0.24
Stimulus 3:Self-Guided GenAI	-0.36	0.19	-0.73 - 0.02
Stimulus 4:Self-Guided GenAI	-0.22	0.19	-0.6 - 0.16
Stimulus 5:Self-Guided GenAI	-0.21	0.2	-0.59 - 0.18
Stimulus 6:Self-Guided GenAI	-0.34	0.2	-0.73 - 0.05
Stimulus 7:Self-Guided GenAI	-0.39	0.19	-0.77 - -0.02

Stimulus 8:Self-Guided GenAI	-0.4	0.19	-0.78 - -0.02
Stimulus 9:Self-Guided GenAI	-0.28	0.19	-0.65 - 0.09
Stimulus 11:Self-Guided GenAI	-0.46	0.19	-0.84 - -0.09
Stimulus 12:Self-Guided GenAI	-0.16	0.19	-0.52 - 0.21
Stimulus (1) x Rater Type (Human)			
Stimulus 2:GPT	-0.14	0.03	-0.2 - -0.08
Stimulus 3:GPT	0.03	0.03	-0.03 - 0.1
Stimulus 4:GPT	0.09	0.03	0.03 - 0.16
Stimulus 5:GPT	-0.24	0.03	-0.3 - -0.18
Stimulus 6:GPT	-0.17	0.03	-0.23 - -0.11
Stimulus 7:GPT	0	0.03	-0.06 - 0.06
Stimulus 8:GPT	0.02	0.03	-0.04 - 0.08
Stimulus 9:GPT	-0.25	0.03	-0.31 - -0.18
Stimulus 10:GPT	0.04	0.04	-0.04 - 0.12
Stimulus 11:GPT	0.08	0.03	0.02 - 0.15
Stimulus 12:GPT	-0.05	0.03	-0.12 - 0.01
Stimulus 2:Guided-GPT	0.04	0.03	-0.02 - 0.1
Stimulus 3:Guided-GPT	0.17	0.03	0.1 - 0.23
Stimulus 4:Guided-GPT	0.16	0.03	0.1 - 0.22
Stimulus 5:Guided-GPT	0.05	0.03	-0.02 - 0.11
Stimulus 6:Guided-GPT	0.02	0.03	-0.04 - 0.09
Stimulus 7:Guided-GPT	0.14	0.03	0.08 - 0.2
Stimulus 8:Guided-GPT	0.15	0.03	0.08 - 0.21
Stimulus 9:Guided-GPT	0.01	0.03	-0.05 - 0.08
Stimulus 10:Guided-GPT	0.13	0.04	0.05 - 0.21
Stimulus 11:Guided-GPT	0.18	0.03	0.12 - 0.25
Stimulus 12:Guided-GPT	0	0.03	-0.06 - 0.07
Category (Visual Artists) x Rater Type (Human)			
Non Artists:GPT	0.34	0.02	0.3 - 0.37
Human Inspired GenAI:GPT	0.54	0.02	0.5 - 0.58
Self-Guided GenAI:GPT	0.62	0.02	0.58 - 0.65
Non Artists:Guided-GPT	0.12	0.02	0.08 - 0.15
Human Inspired GenAI:Guided-GPT	-0.14	0.02	-0.17 - -0.1
Self-Guided GenAI:Guided-GPT	-0.25	0.02	-0.29 - -0.21

Random effects

Image (Intercept)

Model fit

R2

t	p value
35.52	< .001
0.03	0.972
1.23	0.219
1.05	0.294
0.28	0.781
1.67	0.096
1.75	0.08
0.28	0.777
0.92	0.358
1.82	0.07
2.52	0.012
0.65	0.517
-1.93	0.053
-3.82	< .001
-4.33	< .001
-11.87	< .001
-1.31	0.189
0.49	0.622
-0.29	0.774
-0.31	0.753
-0.07	0.94
-1.05	0.293
-1.55	0.121
-0.26	0.798
-0.89	0.374
-0.53	0.595
-0.43	0.67
-0.29	0.769
0.71	0.475
-1.32	0.186
0.09	0.931
2.51	0.012
-0.48	0.632
0.84	0.4
1.46	0.145
1.07	0.286
0.78	0.437
-0.01	0.993
-0.66	0.512
-1.88	0.061
-1.11	0.266
-1.05	0.295
-1.72	0.086
-2.04	0.042

-2.08	0.038
-1.46	0.144
-2.42	0.016
-0.83	0.404
-4.43	< .001
0.98	0.329
3.01	0.003
-7.38	< .001
-5.35	< .001
-0.02	0.987
0.62	0.534
-7.61	< .001
1.03	0.302
2.54	0.011
-1.64	0.101
1.31	0.19
5.06	< .001
5.18	< .001
1.4	0.161
0.71	0.477
4.39	< .001
4.61	< .001
0.38	0.705
3.23	0.001
5.71	< .001
0.02	0.985
18.39	< .001
27.68	< .001
31.51	< .001
6.47	< .001
-6.95	< .001
-12.63	< .001

Variance	S.D.
0.203	0.45

Marginal	Conditional
0.11	0.31
